# Trends in Rescue and Rehabilitation of Marsupials Surviving the Australian 2019–2020 Bushfires

**DOI:** 10.3390/ani14071019

**Published:** 2024-03-27

**Authors:** Holly R. Cope, Clare McArthur, Rachael Gray, Thomas M. Newsome, Christopher R. Dickman, Aditi Sriram, Ron Haering, Catherine A. Herbert

**Affiliations:** 1 Sydney School of Veterinary Science, The University of Sydney, Sydney, NSW 2006, Australia; holly.cope@sydney.edu.au (H.R.C.); rachael.gray@sydney.edu.au (R.G.); 2 School of Life and Environmental Sciences, The University of Sydney, Sydney, NSW 2006, Australia; clare.mcarthur@sydney.edu.au (C.M.); thomas.newsome@sydney.edu.au (T.M.N.); chris.dickman@sydney.edu.au (C.R.D.); 3 New South Wales Department of Climate Change, Energy the Environment and Water, National Parks and Wildlife Service, Parramatta, NSW 2150, Australia

**Keywords:** koala, kangaroo, possum, wildlife, megafire, wildfire, bushfire, disaster, Black Summer, fate, survival, hospitalisation, mortality, release rate

## Abstract

**Simple Summary:**

The 2019–2020 Australian bushfire season had a devastating impact on native fauna. It was estimated that 3 billion animals were affected by the fires, but there are few accounts of the species or numbers of animals rescued and rehabilitated post-fire. We reviewed rescue, triage, rehabilitation and release reports for marsupials from two regions: the state of New South Wales (NSW) and Kangaroo Island, South Australia. In NSW, only 889 marsupial rescues were reported, despite estimates of 46.8 million marsupials inhabiting the fire zones. Three marsupial groups dominated the rescue statistics: kangaroos and wallabies (*n* = 458), koalas (*n* = 204), and possums (*n* = 162), with smaller numbers of other marsupial species. The probability of survival and release was lowest for kangaroos and wallabies (15% ± 4%) compared with koalas (47% ± 4%) and possums (55% ± 10%). Injury type was a significant predictor of survival for all groups, with malnourished/moribund animals or those with traumatic injuries less likely to survive. In both jurisdictions, koalas were over-represented, and possums under-represented, in rescue statistics relative to baseline population densities and pre-fire wildlife rescue trends. Triage decisions also varied by species, with koalas more likely to enter care, whereas other species were more likely to be euthanised at triage. Koalas were more likely to die during rehabilitation, with 73% dying or being euthanised between day 1 and 30 post-rescue, representing a potential welfare concern. These species differences in presentation post-fire warrant further investigation, as do the differences in triage, survival and release outcomes. These data highlight the need for detailed record keeping and data sharing, and the development of consistent and evidence-based triage, treatment and euthanasia guidelines for all species.

**Abstract:**

The 2019–2020 Australian bushfire season had a devastating impact on native wildlife. It was estimated that 3 billion native animals were impacted by the fires, yet there are few estimates of the number of animals that were rescued and rehabilitated post-fire. Focusing on the state of New South Wales (NSW) and Kangaroo Island, South Australia, we used a case study approach to determine the number of marsupials that were reported rescued due to the 2019–2020 bushfires in these areas and analysed species-specific trends in rescue and release success. In NSW, we found 889 reports of fire-affected marsupials in 2019–2020, mostly comprising kangaroos and wallabies (macropods; *n* = 458), koalas (*n* = 204), and possums (*n* = 162), with a smaller number of wombats (*n* = 43) and other marsupial species. Most reports of fire-affected marsupials occurred 6–8 weeks after fire ignition, and there was no difference in temporal frequency of rescues between marsupial groups. For the three main groups, the probability of survival and subsequent release differed, with macropods having the lowest probability of release after rescue (0.15 ± 0.04) compared to koalas (0.47 ± 0.04) and possums (0.55 ± 0.10). The type of injury was the main predictor of survival during rehabilitation for all three marsupial groups, with those malnourished/moribund or with traumatic injuries less likely to survive rehabilitation. Death or euthanasia occurred on the day of rescue for 77% of macropods, 48% of possums and 15% of koalas. Koalas most often died during rehabilitation rather than on the day of rescue, with 73% either dying or being euthanised between day 1 and 30 post-rescue, representing a potential welfare concern. On Kangaroo Island, koalas were the most frequently rescued marsupial species; most euthanasia cases and deaths occurred in a hospital, whereas other marsupials were mostly euthanised at triage. In both jurisdictions, koalas were over-represented while possums were under-represented relative to baseline population densities and wildlife rescue trends in the years before the 2019–2020 bushfires. These species differences in presentation post-fire warrant further investigation, as do the differences in triage, survival and release outcomes. It is hypothesised that the high intensity and large scale of the 2019–2020 fires impeded marsupial fire evasion tactics, as evidenced by the small number of animals found for rescue, and the differing rates of presentation relative to underlying population densities for the main marsupial groups. Based on our findings, there is a need for detailed record keeping and data sharing, development of consistent and evidence-based triage, treatment and euthanasia guidelines and deployment of trained wildlife emergency rescue teams with advanced search techniques to minimise animal suffering where safe to do so.

## 1. Introduction

The Australian bushfire season of 2019–2020, sometimes referred to as the “Black Summer” of fires, had a major impact on native flora and fauna [1,2,3]. An estimated 3 billion animals were impacted, directly or indirectly [4] when 15,000 wildfires burnt an estimated 19 million ha of forest and woodland in south-eastern Australia [5]. The 2019/20 bushfires, along with several other Australian and international environmental disasters in recent years, have been linked with climate change [6,7,8,9,10]. As climate-fuelled extreme weather events continue to increase in frequency, severity and scale, there will be major consequences for wildlife [6,11]. Hence, we need to identify and understand the factors affecting wildlife survival post-fire, both in the landscape and during rescue, rehabilitation and release efforts, so we can develop management strategies to improve outcomes for wildlife affected by these major threats.

Wildlife rescue and rehabilitation are increasingly recognised as important components of emergency disaster responses [12,13]. While we have estimates of the number of native animals killed or displaced during the 2019/20 fires [4], published accounts of the magnitude and outcomes of bushfire-associated rescue and rehabilitation have been scant, except for recent estimates for the number of koalas rescued in New South Wales (NSW) [14] and triage assessment findings for fire-affected koalas on Kangaroo Island [15]. Lunney et al. [14] found that 209 koalas were rescued in NSW, but we do not know how their rescue rates compare with other species, or their magnitude relative to their pre-existing population densities.

It has been acknowledged that there was a significant gap in disaster preparedness among wildlife rescue and rehabilitation groups in Australia prior to the 2019/20 fires [16,17,18] and there is a need to formally include wildlife rescue in state emergency response protocols with greater consistency and collaboration [19,20,21,22,23]. Therefore, knowledge of the presentation patterns and outcomes of wildlife rescue and rehabilitation efforts during the 2019/20 fires represents an important baseline dataset that can be used to improve emergency response protocols within the sector. 

Here we determine the species-specific susceptibility of marsupials to bushfire injury and subsequent rescue and evaluate factors influencing the probability of release from rehabilitation in the wake of the 2019/20 fires in south-eastern Australia. We focus on two areas where there was consistent data reporting: the state of NSW and Kangaroo Island, South Australia (SA). Using a case-study-based approach, we collate and analyse wildlife rescue and rehabilitation records from the 2019/20 fires to determine the following: (i) the species-specific frequency of reporting to rehabilitation organisations and subsequent entry of animals into rehabilitation; (ii) the types of injuries, length of rehabilitation, temporal patterns and outcomes for different marsupial species over the study period; (iii) the factors that correlate with survival to release for each species; and (iv) the species-specific presentation rates compared with the underlying population density and non-fire-related rescue rates in the preceding years. 

NSW was one of the most significantly impacted states during the 2019/20 bushfires [24,25]. The NSW National Parks and Wildlife Service (NPWS) has systematically collected wildlife rehabilitation statistics during the past decade [26], allowing for meaningful comparisons of trends in rescue and rehabilitation due to the fires to be made at a large scale. We utilised the NPWS wildlife rehabilitation database to present an account of the rescue and rehabilitation of marsupials in NSW with comparisons to nominal baseline figures for fire-related and non-fire-related rescues in the previous four years. Kangaroo Island (4505 km^2^) was also significantly impacted when about half of the island burnt in the 2019/20 fires [27]. This island is inhabited by large populations of three of the most rescued marsupial groups: kangaroos and wallabies (hereafter referred to as “macropods”), possums and gliders (hereafter referred to as “possums”) and koalas, with some indicative baseline density data also available. Therefore, this island provides a useful case study for analysis of trends in rescue rates by species relative to their underlying population density. 

Factors affecting wildlife survival during rehabilitation and following release are species- and context-specific [28]; hence, it was hypothesised that survival outcomes would vary between species, injury types and fire severity zones. The main marsupial species rescued due to fires in NSW include macropods, possums and koalas [29]. These groups have differing life-history traits (i.e., gregarious, crepuscular, terrestrial macropods versus solitary, nocturnal, arboreal possums and koalas) which could affect their survival rates. Therefore, we hypothesised that rescue rates of marsupials would vary between species independently of their relative abundance within the ecosystem, due to differing life-history strategies influencing their movement behaviour [30], ability to shelter or escape from fires [4,31] and their visibility and accessibility to rescuers. We also predicted that individuals with traumatic injuries would have the worst survival outcomes (irrespective of species) [28] and that high fire severity zones would have the lowest survival outcomes. 

## 2. Materials and Methods

### 2.1. NSW Wildlife Rehabilitation Data Collation

Wildlife rescue and rehabilitation data from 43 licensed volunteer wildlife rehabilitation providers were supplied by NSW National Parks and Wildlife Service (NPWS) with records from 2015/16 to 2019/20 including species rescued, reason for rescue, rescue and release location coordinates, and fate. Records from the Wildlife Information Rescue and Education Service (WIRES), the largest wildlife rehabilitation organisation in NSW, were collated as part of the NPWS database, and WIRES provided direct access to their database so that call transcripts could be viewed for further details of marsupial rescues in 2019/20. Rescue and rehabilitation summary figures for all wildlife species from 2015/16 through 2019/20 were accessed via the NSW Wildlife Rehabilitation Dashboard [29]. Wildlife rescue records are reported to NPWS in Australian financial year units (1st July to 30th June), and this format was retained in the current analysis. This format also ensured that the entirety of the 2019/20 fire season was included, as the last of the 2019/20 bushfires were extinguished by heavy rain in March 2020. 

Not all rescue records constitute an animal taken into rehabilitation. For example, some “rescue” records do not involve actual rescue of the animal but may instead only require provision of advice to the caller, or the animal may not be able to be located for rescue. Hence, “rehabilitation” admission records are a subset of reported records. For marsupials, 65% of records reported admission to rehabilitation in 2015/16, and this reduced to 55% in 2019/20 (Table S1), indicating an increase in the proportion of calls that were resolved without the animal coming into human care. Animals that were found dead on arrival by rescuers were not classed as admissions to rehabilitation; however, animals that were euthanised at the time of rescue could not be separated from those euthanised several hours after rescue based on available data and were therefore classed as admissions and included in the fate of “euthanised/died in care” for analysis. 

Species were categorised into seven marsupial groups: bandicoots (Peramelidae); bettongs and potoroos (Potoroidae); dasyurids (antechinuses, dunnarts, quolls, phascogales, planigales; Dasyuridae); koala (*Phascolarctos cinereus*: Phascolarctidae); macropods (including kangaroos, wallabies and undifferentiated kangaroos/wallabies; Macropodidae); possums and gliders (hereafter referred to collectively as possums; Phalangeridae, Pseudocheiridae and Petauridae); and wombats (Vombatidae). Injury types were categorised as follows: abandoned/orphaned; heat stress/dehydrated; malnourished/moribund; trauma (including injuries, respiratory difficulty and burns); other (disease, cold stress/hypothermia, abnormal behaviour, concussed, nothing apparent, no apparent distress, skin problem, shock); and unknown/unclassified (treated as missing data in statistical analyses). Burns and injuries were categorised together under trauma in our analysis due to suspected inconsistencies between groups and years and the potential that burns were recorded as injuries in some cases. We calculated the length of stay (LOS) in rehabilitation as the date of fate minus the date of rescue.

### 2.2. Data Analysis

#### 2.2.1. Rates of Presentation and Fate during and before the 2019/20 Fires

Descriptive statistics were calculated for all wildlife species to determine the frequency of entry into rehabilitation due to all causes for rescue and fire-related rescues over the periods 2015/16–2018/19 and 2019/20. The 2015/16–2018/19 period provides a baseline for all rescues, along with fire-related rescues specifically, prior to the 2019/20 bushfires. A linear regression was used to test for a relationship between the number of wildlife rehabilitation volunteers, as listed in the NSW Wildlife Rehabilitation Annual Report 2021–22 [32], and the total yearly wildlife rescues. Descriptive statistics were also calculated for fire-related rescues of marsupials in 2019/20 to summarise the frequency of rescue by marsupial group, sex, age, fire severity zones, distance from the fire extent at the location of rescue (m), and days since fire ignition when rescued. A contingency chi-squared test was used to test for a difference in the proportions of rescues attributed to macropods, koalas, possums and wombats in 2015/16–2018/29 versus 2019/20, and for a difference in the proportions of rescues attributed to each fate in 2015/16–2018/29 versus 2019/20.

#### 2.2.2. Predictors of Fate for Fire-Affected Marsupials

A general linear model with binomial distribution and logit link function was used to test for an effect of marsupial group on the probability of release. The probability of release was calculated as a binomial, with zero (0) representing the fate of euthanised/died in care and one (1) representing the fate of released/relocated. Therefore, the release probability does not include fates of animals classified under “advice provided”, “could not locate or catch”, “dead prior to rescuer arriving”, “escaped”, “in care”, “left and observed”, “permanent care”, “record of sighting”, “referred to or resolved by external organisation”, “rehomed”, “returned”, “transferred” and “unknown”. The marsupial groups including bandicoots, bettongs and potoroos, dasyurids and wombats had fewer than 20 release or euthanasia records and were therefore excluded from the analysis. Macropods, koalas and possums had sufficient records for analysis.

Each marsupial group was then assessed separately using a general linear model with binomial distribution and logit link function to determine the effect on probability of release of injury type (abandoned/orphaned, heat stress/dehydrated, malnourished/moribund, trauma or other), age (juvenile or adult), sex (male or female), fire severity (nonburnt, medium, high or extreme), rescue within a fire zone (in or out), fire site and days since fire ignition when rescued. Each predictor was analysed in a separate model due to highly unbalanced data resulting from large amounts of missing data, making a comparison of models incorporating different sets of variables by Akaike’s Information Criterion (AIC) methods invalid. Tukey’s HSD was used to test for significant differences between pairwise comparisons of levels of factors that were significant in the general linear model.

#### 2.2.3. Sex and Age of Fire-Affected Marsupials

We established if there were any sex or age biases in marsupials rescued due to fires in 2019/20, and whether this matched trends in fire rescues in the preceding years (2015/16–2018/19) or rescues for any cause across both time periods. For each marsupial group with sufficient records for analysis (macropods, koalas and possums; excludes those with less than 20 records in any year), a general linear model with Poisson distribution and logit link function was used to test for an effect of sex (male or female) and age (juvenile or adult) on the annual number of rescues recorded (2015/16–2019/20). A contingency chi-squared test with Yates’ continuity correction was then used to test for a change in proportions of known sex and age groups between 2019/20 and the preceding years (2015/16–2018/19). Age categories varied in use between years, so Adult additionally includes “Geriatric” and Juvenile additionally includes “Sub-adult” and “Young”.

#### 2.2.4. Days since Fire Ignition When Reported

Each fire-related rescue record was allocated to a 2019/20 wildfire site using the NPWS Fire History—Wildfires and Prescribed Burns [33] layer in ArcMap 10.8. Days since fire ignition at rescue were calculated for each fire-affected marsupial in 2019/20 by subtracting the date of fire site ignition [33] from the date of encounter. It is important to note that fire boundaries are dynamic and vary over time.

A general linear mixed model with fire site as a random effect was used to test for an effect of marsupial group (macropods, koalas and possums) on the days since fire ignition when the fire-affected animal was reported. All analyses were undertaken in R version 4.0.5 [34] and considered significant at *p* < 0.05.

#### 2.2.5. Length of Stay in Rehabilitation for 2019/20 Fire-Affected Marsupials

Length of stay (LOS) in rehabilitation was evaluated separately for rescues with a fate of released/relocated and euthanised/died in rehabilitation (LOS analysis excludes marsupials that were dead on arrival, were not admitted for rehabilitation, were still in care at the time of reporting, or had an unknown fate). For each fate, a general linear model was used to test for a difference in LOS between macropod, koala and possum groups. For each marsupial group, the effects on LOS of injury type, age, sex, fire severity, rescue within a fire zone, fire site and days since fire ignition when rescued were tested separately for those that were released and those that died. Fire site was tested as a random effect, but due to issues with singularity, it was tested as a fixed effect. 

### 2.3. Spatial Analysis

NSW wildlife rescue locations were plotted in ArcMap 10.8 and overlaid with fire extent (National Indicative Aggregated Fire Extent 1 July 2019 to 22 June 2020 [35]), fire severity (Fire Extent and Severity Mapping (FESM) 2019/20 [36]) and IBRA region (Australian Bioregion; IBRA version 5.1 [37]) layers. Data were extracted for each rescue record to determine whether rescues were located within the fire zone, the fire severity in the location of rescue (for rescues within the fire zone), the distance that animals were rescued from the edge of the fire zone (for rescues outside the fire zone) and the relevant IBRA bioregion (all rescues). For distance calculations, only records with spatial coordinates with an accuracy of less than 500 m were used. 

### 2.4. NSW Baseline Species Density Data

Baseline animal density data for NSW were extracted from the work of van Eeden et al. [4] for macropods and possums and from the work of Adams-Hosking et al. [38] for koalas, focusing on the four most impacted IBRAs: NSW North Coast, South East Corner, South Eastern Highlands and the Sydney Basin. 

### 2.5. NSW Marsupial Rehabilitation Records in Context

#### 2.5.1. Completeness of NSW Fire Rescue Records

In 2019/20, there were 37,076 marsupial rescue records in the NPWS database. Of these, 22,966 had an unknown cause for rescue. As an indication of the proportion of “missed” fire-related marsupial records, the WIRES rescue database (a subset of the NPWS rescue database), was searched for records with an unclassified cause for rescue for three commonly rescued marsupial species in a four-month period between 1 November 2019 and 29 February 2020 to capture the height of the bushfire season in NSW (noting there were also major events in October 2019 in northern NSW). Individual call sheets were accessed, and transcripts were used to determine whether the rescue was related to wildfire. 

#### 2.5.2. Marsupial and Fire Records in the Broader Context of All Species and Events

Given that marsupials are not the only species that are brought into care during bushfires, we also present descriptive statistics for marsupials as a proportion of all wildlife rehabilitation records before and during the 2019/20 bushfires. This allows for the marsupial rescue activity during the 2019/20 bushfires to be placed within the broader context. 

### 2.6. Comparison with Kangaroo Island 2019/20 Fires

#### 2.6.1. Frequency of Rescues and Triage Outcomes

Triage data for 635 native animals rescued on Kangaroo Island, South Australia, in 2019/20 were provided by Zoos SA from the Kangaroo Island Wildlife Park Hospital. Data collection details and triage assessments for koalas have been described by Dunstan et al. [15]. These data were used to describe the rates of presentation for the four most common marsupial species on the island: tammar wallabies (*Notamacropus eugenii*, hereafter wallaby), Kangaroo Island western grey kangaroos (*Macropus fuliginosus fuliginosus*), koalas and brushtail possums (*Trichosurus vulpecula*). 

#### 2.6.2. Baseline Population Densities

On Kangaroo Island, population estimates for tammar wallabies, western grey kangaroos and koalas were extracted from survey work conducted by South Australia Department for Environment and Water prior to the 2019/20 bushfires on Kangaroo Island [39,40], with estimates for brushtail possums from the work of van Eeden et al. [4].

## 3. Results

### 3.1. Completeness of NSW Fire Rescue Records

We found that 37 of 999 unclassified records in the WIRES rescue database detailed fire-related injuries for eastern grey kangaroos (*Macropus giganteus*; *n* = 16 of 371; plus 5 more that were possibly fire-related), common brushtail possums (*Trichosurus vulpecula*; *n* = 14 of 320) and common ringtail possums (*Pseudocheirus peregrinus*; *n* = 7 of 308). This represents 3.7% of unclassified records that should have been allocated to the “Event—Fire” cause for rescue for these three key species. Therefore, while the database used for this study is an incomplete account of all marsupials rescued due to bushfires in 2019/20, the number of missing records is relatively small and unlikely to impact the overall trends reported.

### 3.2. Rates of Presentation and Fate during and before the 2019/20 Bushfires

In 2019/20, there were 889 marsupial records from 29 different species reported in NSW with the cause listed as “Event—Fire” (referred to as “fire-affected” or “rescues due to fire”, hereafter), and 76% of these animals were admitted to rehabilitation (the remainder being sightings or where advice was provided; Table S2). 

**Figure 1:**
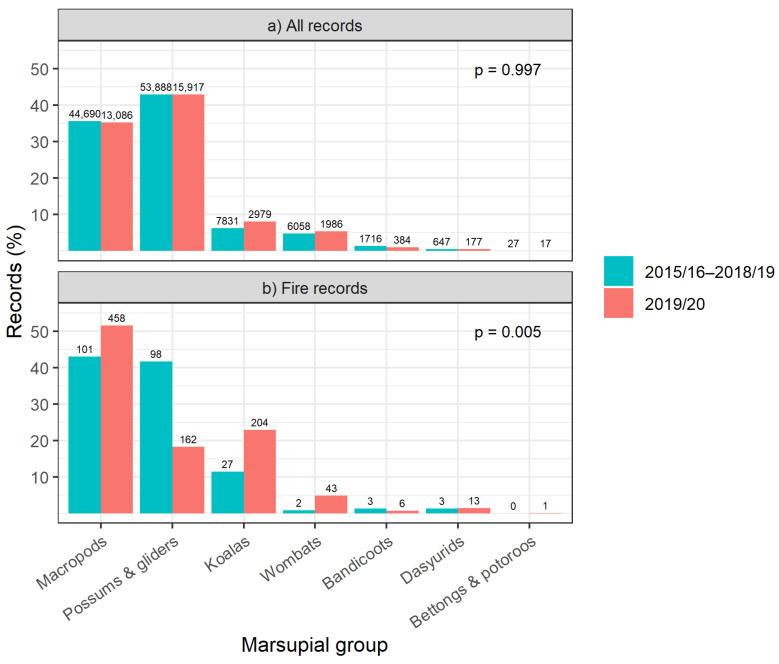
Marsupial group as a proportion (%) of (**a**) all marsupial records and (**b**) fire-affected marsupial records for the years 2015/16–2018/19 (blue columns; *n* = 125,439 for all records; *n* = 235 for fire records) and 2019/20 (red columns; *n* = 37,076 for all records; *n* = 889 for fire records). Record counts (*n*) are presented above columns for each taxonomic group.

The rates of presentation for different marsupial groups rescued for any cause in 2019/20 did not differ from previous years (χ^2^_5_ = 0.32, *p* = 0.997; Figure 1a), but the proportions of fire-affected records for each marsupial group differed significantly in 2019/20 compared with fire events in previous years (χ^2^_5_ = 16.63, *p* = 0.005). Fire-affected macropod, koala and wombat records were proportionately higher in 2019/20 compared to the period 2015/16–2018/19, while possums were proportionately lower (Figure 1b). Macropods were the most frequently fire-rescued species in 2019/20, while in previous years, macropods and possums were reported in similar numbers due to fire, and possums were most often reported due to any cause (Figure 1). 

**Figure 2:**
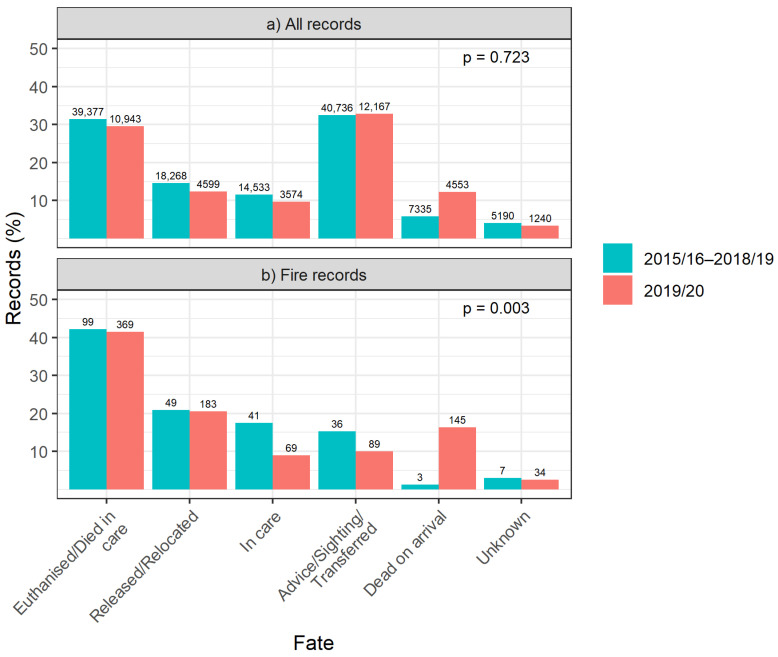
Fate as a proportion (%) of (**a**) all marsupial records and (**b**) fire-affected marsupial records in 2015/16–2018/19 (blue columns) and 2019/20 (red columns). Record counts (*n*) are presented above columns. The fate of “In care” indicates that the animal was still in care at the time of reporting, and the fate of “Dead on arrival” indicates that the animal died between the time it was reported and when a rescuer arrived.

The fate of fire-affected marsupials differed significantly between 2019/20 and previous years (χ^2^_5_ = 17.7, *p* = 0.003), yet there was no difference between these time periods when looking at all causes for rescue (χ^2^_5_ = 2.8, *p* = 0.723; Figure 2). In 2015/16–2018/19, 51% of fire-affected marsupials admitted to rehabilitation were euthanised or died in care, 25% were released or relocated and 21% were still in care at the time of reporting (*n* = 196 admitted of 235 records). Macropods and possums were most often reported, and their fate was frequently euthanised/died in care (see Table S3 for fates by marsupial group). A greater proportion of marsupials were found dead on arrival of a rescuer in 2019/20 compared to 2015/16–2018/19 (χ^2^_5_ = 17.7, *p* = 0.003; Figure 2), and macropods were reported more frequently than possums (*n* = 458 versus *n* = 162, respectively; Table 1). Overall, 56% of fire-affected marsupials admitted to rehabilitation in 2019/20 were euthanised or died in care, 28% were released or relocated and 11% were still in care at the time of reporting (*n* = 655 admitted of 889 records; *n* = 145 dead on arrival; Table 1). Macropods and koalas were most frequently rescued, and their fate was mostly euthanised/died in care (75% and 43%, respectively; Table 1), followed by possums, which were mostly released (48%). Wombats had a very low release rate (6.3%), but this was due to the large proportion still in care at the time of reporting, rather than a reflection of the true release rate. 

#### 3.2.1. Types of Injuries for Fire-Affected Marsupials

Most (66%) injuries of fire-affected marsupials in 2019/20 were categorised as trauma (including burns, injuries to various body parts and respiratory difficulty; see Table S4 for injury types by marsupial group). Abandonment and orphaning, heat stress and dehydration, malnourishment and moribundity, and other less common injury types made up the remainder of the known causes of injury. The injury types in 2015/16–2018/19 followed a similar trend in proportions to those in 2019/20 (χ^2^_8_ = 11.4, *p* = 0.182), although burns were recorded more frequently than injury in the trauma category, being 62% of trauma cases in 2015/16–2018/19 versus 38% in 2019/20. 

#### 3.2.2. Predictors of Fate for Fire-Affected Marsupials

**Figure 3:**
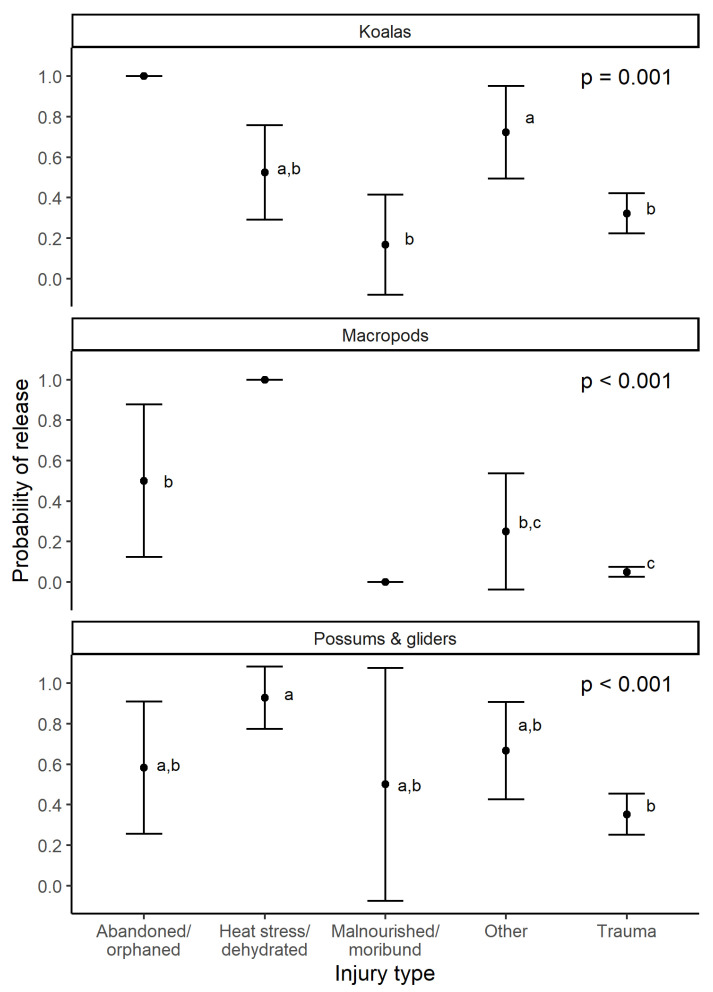
Mean ± 95% CI probability of release (0 = euthanised/died in care, 1 = released/relocated) of fire-affected marsupials in 2019/20 for koalas, macropods, and possums and gliders, by injury type. Superscript letters indicate injury types for each marsupial group that are significantly different based on pairwise comparisons.

Of all fire-affected marsupials successfully rescued in 2019/20 (i.e., excluding those with fates of found dead on arrival of rescuer, advice/sighting/transferred, in care and unknown), macropods were significantly less likely to be released than koalas or possums (probability of release, mean ± s.e.: macropods = 0.15 ± 0.04, koalas = 0.47 ± 0.04, possums = 0.55 ± 0.06; LR χ^2^ = 81.62, d.f. = 2, *p* < 0.001, deviance explained = 12.4%). Injury type was a significant predictor of release for each marsupial group (macropods: LR χ^2^_4_ = 69.4, *p* < 0.001; koalas: LR χ^2^_4_ = 13.5, *p* = 0.009; possums: LR χ^2^_4_ = 23.8, *p* < 0.001; Figure 3). Age was a significant predictor of release for macropods (LR χ^2^_1_ = 4.3, *p* = 0.037), with juvenile macropods having a significantly greater probability of release than adults (*p* = 0.010; mean ± s.e. = 0.68 ± 0.07 and 0.12 ± 0.03, respectively). Age was not significant for possums and was approaching significance for koalas (LR χ^2^_1_ = 3.7, *p* = 0.056), but likely because of juveniles being largely abandoned/orphaned, and thus, captured by injury type. Fire severity (LR χ^2^_4_ = 13.0, *p* = 0.011) and days since fire ignition when rescued (LR χ^2^_1_ = 6.0, *p* = 0.014) were significant predictors for possums. See Table S5 for full general linear model output. 

#### 3.2.3. Sex and Age of Fire-Affected Marsupials

**Figure 4:**
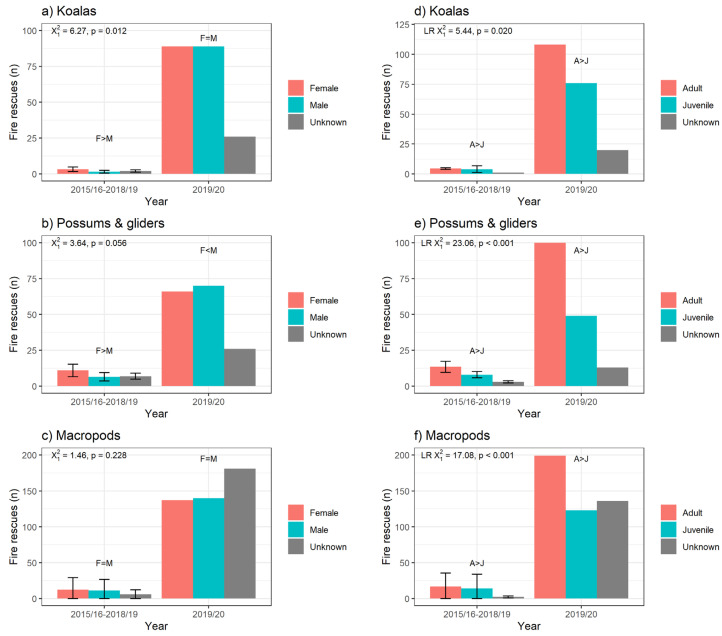
Number of fire-affected marsupial rescue records in 2015/16–2018/19 and 2019/20 for koalas, possums and macropods by sex (**a**, **b** and **c**, respectively) and by age (**d**, **e** and **f**, respectively). The data for the period 2015/16–2018/19 are presented as yearly mean ± SD, while the data for the year 2019/20 are absolute numbers. LR chi-squared analyses are testing for an overall difference in proportions of ages (where no change occurred between years), and chi-squared analyses are testing for a change in relative proportions of sexes between years.

Of the macropods, koalas and possums rescued, sex was left blank or recorded as unknown for 56% of rescues for all causes and 27% of fire-related rescues from 2015/16 to 2019/20. Male macropods and koalas were rescued more often per year than females for all causes (LR χ^2^_1_ = 186.79, *p* < 0.001, and LR χ^2^_1_ = 21.71, *p* < 0.001, respectively), whereas possums had a bias towards females (LR χ^2^_1_ = 128.35, *p* < 0.001), and these trends remained when comparing years before (2015/16–2018/19) and during the 2019/20 bushfires (see Table S6 for full model output). No marsupial group had an overall bias towards either sex for fire-related rescues (Figure 4a–c). Age was left blank or unknown for 38% of rescues for all causes and 18% of fire-related rescues from 2015/16 to 2019/20. More juvenile macropods and possums were rescued per year than adults due to all causes for rescue (LR χ^2^_1_ = 257.11, *p* < 0.001, and LR χ^2^_1_ = 1096.9, *p* < 0.001, respectively), whereas more adult koalas were rescued than juveniles (LR χ^2^_1_ = 2464.7, *p* < 0.001; see Table S6 for full model output). These trends remained the same when comparing years before and during the 2019/20 bushfires. More adult koalas, macropods and possums were rescued per year than juveniles due to fires (Figure 4d–f). There was no change in these biases in the years before or during the 2019/20 bushfires. 

#### 3.2.4. Days since Fire Ignition When Reported

**Figure 5:**
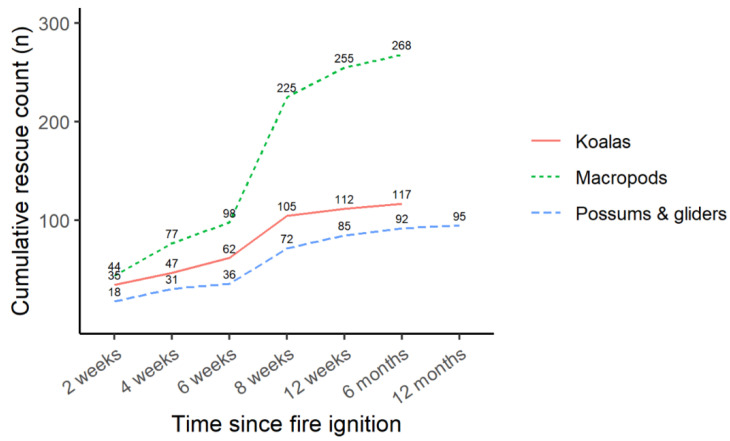
Cumulative number of koalas, macropods, and possums rescued due to fires in 2019/20 (*n* = 480 with accurate dates of rescue and fire ignition, excluding those found dead) over 12 months following ignition of the fire at the relevant rescue location. Cumulative *n* values are presented above each line.

The largest number of fire-affected marsupial rescues occurred 6–8 weeks post-fire ignition for all species (Figure 5, Table S7). The second largest number of rescues occurred within two weeks of fire ignition. There were 95 records of marsupials found dead within 2 weeks of fire ignition out of a total of 130 found dead overall. Possums and gliders were rescued later than macropods and koalas after fire ignition (χ^2^_2_ = 9.3, *p* = 0.010). Including the random effect of fire site improved the model (AIC = 6049.3 versus AIC = 6231.5 with no random effect).

#### 3.2.5. Length of Stay in Rehabilitation for 2019/20 Fire-Affected Marsupials

For marsupials that were released or relocated (*n* = 183 with accurate date of fate), the mean length of stay (LOS) in rehabilitation was 65.8 days (SD 57.9; range 0–306 days), whereas for those that died or were euthanised in care (*n* = 369 with accurate date of fate), the mean LOS in rehabilitation was 8.2 days (SD 24.9; range 0–239 days). At the time of reporting, two wombats had been released but did not have dates of fate recorded, and a large number were still in care. 

**Figure 6:**
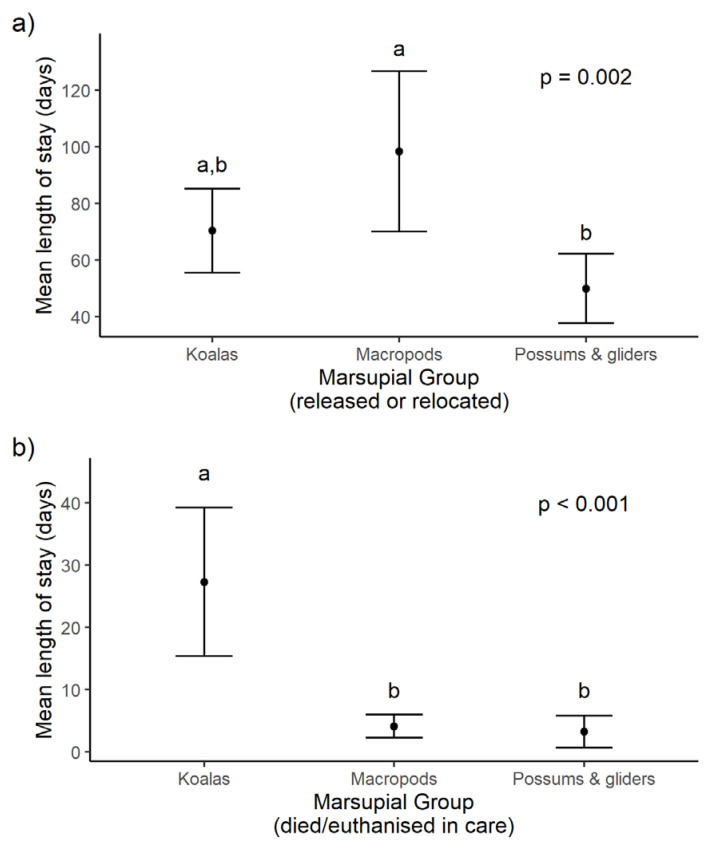
Mean ± 95% CI length of stay (days) of fire-affected koalas, macropods, and possums and gliders that were (**a**) released or relocated (*n* = 183) and (**b**) died or euthanised in rehabilitation after rescue (*n* = 369) in 2019/20. Note the different scales on the *y*-axis between figure (**a**,**b**). Marsupial groups with significantly different lengths of stay are denoted with different superscript letters (a or b) at *p* ≤ 0.001.

There was a significant difference in LOS between macropods, koalas and possums that were released/relocated (F_2,141_ = 6.73, *p* = 0.002; Figure 6a) and those that died or were euthanised in rehabilitation (F_2,322_ = 47.61, *p* < 0.001; Figure 6b). Pairwise comparisons indicate that possums had a shorter LOS until release than macropods (*p* = 0.001). Koalas had a longer LOS until death or euthanasia (27.3 days [SD 47.1]; *p* = 0.533) than both macropods (4.1 days [SD 13.7]; *p* < 0.001) and possums (3.2 days [SD 9.4]; *p* < 0.001), which had similar LOSs. Death or euthanasia occurred on the day of rescue for 77% of macropods (*n* = 161 of 209), 48% of possums (*n* = 26 of 54) and 15% of koalas (*n* = 9 of 62). Koalas more often died during rehabilitation than on the day of rescue, with 73% either dying or being euthanised between day 1 and 30 post-rescue.

#### 3.2.6. Predictors of Length of Stay for Fire-Affected Marsupials

**Figure 7:**
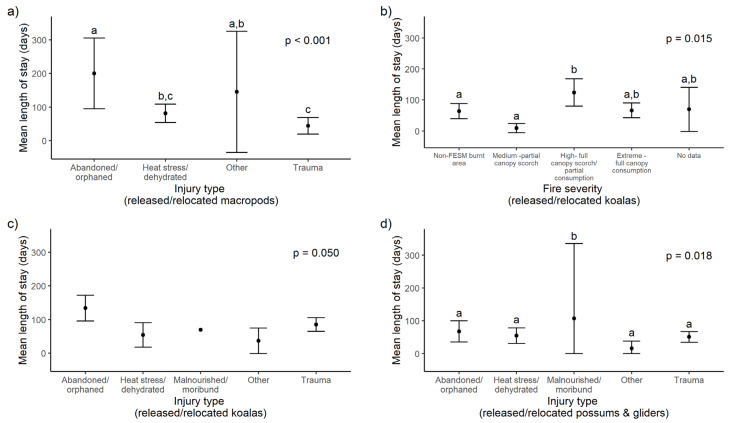
Mean ± 95% CI length of stay (days) relative to significant predictor variables for fire-affected marsupial groups released/relocated in 2019/20: (**a**) macropods by injury type, (**b**) koalas by fire severity, (**c**) koalas by injury type, and (**d**) possums and gliders by injury type that were. Note: The 95% CI for koala injury type malnourished/moribund was wide [−763 and 902] and is not displayed. Superscript letters denote significant differences between categorical variables as determined using pairwise comparisons.

Injury type was a significant predictor of LOS for released or relocated marsupials in all three groups (Figure 7). Age was also a significant predictor for macropods, with juveniles having a longer LOS than adults (*p* = 0.041; 120.0 (SD 83.7) days and 62.5 (SD 36.5) days, respectively). For koalas, age, sex, fire severity (Figure 7b) and fire site were also significant predictors of LOS (Table 2; see Table S8 for full model output). Koalas rescued from high fire severity zones had longer LOS than unburnt and medium burnt zones (Figure 7b). Tukey’s HSD did not detect pairwise differences for koala injury types, although abandoned/orphaned had the longest mean LOS (Figure 7c). Juvenile koalas had longer LOS than adults (*p* < 0.001; 96.3 ± 53.8 days and 45.9 ± 51.9 days, respectively), and female koalas had longer LOS than males (*p* = 0.042; 84.3 ± 56.6 days and 54.4 ± 56.8 days, respectively). Injury type was the only significant predictor for possums, and malnourished/moribund possums had a longer LOS than other causes (*p* = 0.013; Figure 7d).

**Figure 8:**
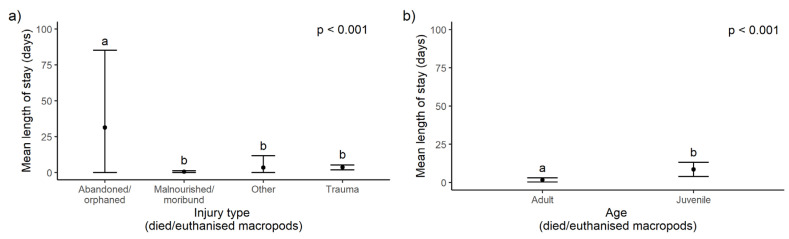
Length of stay (days) for fire-affected macropods and koalas that died or were euthanised in rehabilitation after rescue in 2019/20 relative to the significant predictor variables outlined in Table 3. Macropod mean (±95% CI) length of stay (days) versus (**a**) injury type and (**b**) age. Superscript letters denote significant differences between categorical variables as determined using pairwise comparisons.

For marsupials that died or were euthanised in rehabilitation, the significant predictors of LOS in rehabilitation were injury type (Figure 8a), age (Figure 8b) and fire site for macropods and fire site for koalas, and there were no significant predictors for possums (Table 2; see Table S8 for full model output).

#### 3.2.7. Location of Rescue in Relation to Fire Extent

**Figure 9:**
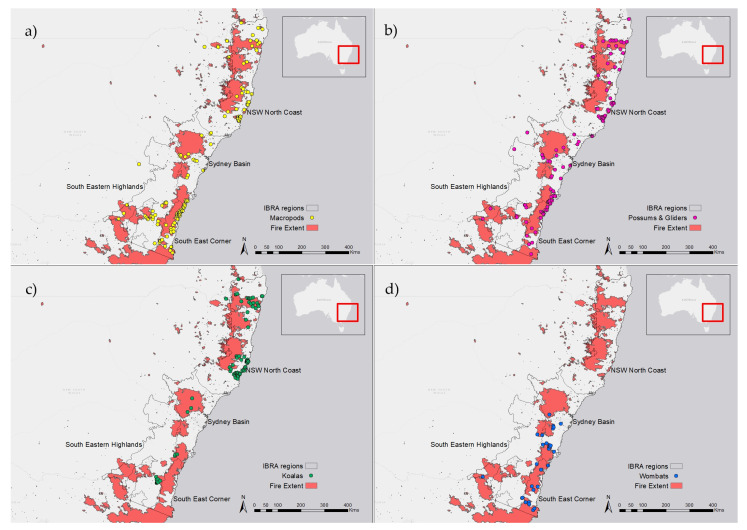
Location of rescue due to 2019–2020 bushfires for (**a**) macropods, (**b**) possums and gliders, (**c**) koalas and (**d**) wombats.

Of the 889 2019/20 bushfire marsupial records, 509 were located within the fire extent (375 locations were only provided as a suburb, so rescues were plotted at the centre of the suburb), and 238 were rescued outside the fire extent (and a further 142 had missing spatial data). Records were generally clustered around the edge of the fireground (Figure 9 and Figure S1). For records located outside the fire extent (and with spatial accuracy <500 m), the average distance to the fire edge was 1.4 km (*n* = 80; s.d. = 2.2 km). Most animals (*n* = 76 of 80) were located within 5 km of a fire, and 52.5% (*n* = 42 of 80) were located within 500 m of a fire. There was no effect of marsupial group (F_2,65_ = 1.0, *p* = 0.373) or injury type (F_3,60_ = 0.1, *p* = 0.97) on the distance from the fire extent when reported. Most reported koalas within the fire zone were in high fire severity zones, while macropods and possums were mostly located in low and moderate severity zones (Table 3). 

#### 3.2.8. Bioregion of Reported Fire-Affected Marsupials in 2019/20

**Figure 10:**
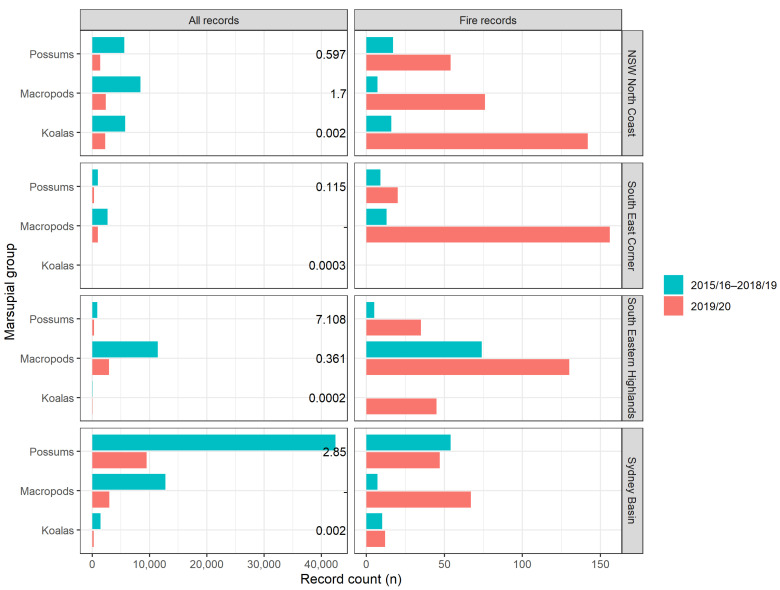
Number of records for possums, macropods and koalas in each of four main IBRA regions (NSW North Coast, South East Corner, South Eastern Highlands, Sydney Basin) with any cause for rescue (all records) and fire-affected animals (fire records) across the years preceding the 2019–2020 bushfires in eastern Australia (2015/16–2018/19; blue columns) and the year of the 2019–2020 bushfires (2019/20; red columns). Density estimates for possums, macropods and koalas are annotated down the centre of the chart (per ha); macropod and possum densities were extracted from van Eeden et al. [4]; koala densities were calculated as mean population size from Adams-Hosking et al. [38] over IBRA size (ha) from DAWE [41].

Most fire-affected macropod (Figure 9a) and possum (Figure 9b) reports in 2019/20 occurred in four NSW IBRAs: NSW North Coast, South East Corner, South Eastern Highlands and the Sydney Basin (Figure 10). Koalas were reported mostly in the NSW North Coast and South Eastern Highlands IBRAs (Figure 9c and Figure 10) and are discussed in detail in the work of Lunney et al. [14]. Wombats were reported in the South East Corner (*n* = 12), South Eastern Highlands (*n* = 14) and Sydney Basin (*n* = 16; Figure 9d). The dasyurids, bandicoots, bettongs and potoroos were all rescued in the NSW North Coast (*n* = 20). 

In all four bioregions, macropods were more frequently reported due to fires than possums in the 2019/20 bushfire season (Figure 10). In three of four bioregions, this was consistent with the trends for all causes for rescue in 2019/20, as well as the pre-fire period (all rescues and fire rescues). However, in the Sydney Basin, possums were rescued in much greater numbers than macropods due to any cause preceding the 2019/20 bushfires (including previous fire events), but macropods were rescued at a rate of 1.5-fold greater than possums due to the 2019/20 bushfires (Figure 10).

Only two of the four IBRAs had published densities for macropods, and possum density varied considerably between the bioregions [4]. Koala densities were low across all IBRAs [38]. In the NSW North Coast, koalas were the most frequently rescued species, even though their estimated density was the lowest and their rates of rescue in the preceding years were comparable to, or lower than, those of the other groups (Figure 10). In the South Eastern Highlands, koalas were rescued at a rate comparable to possums in the 2019/20 bushfires, despite their very low density and scant records preceding the fires and for other causes (Figure 10). In the South East Corner, more macropods were rescued than possums due to any cause before and during the 2019/20 bushfires, despite estimated densities of 0.361 and 7.1, respectively (from van Eeden et al. [4] (Figure 10)). 

### 3.3. Fire-Affected Marsupial Records in Context

#### 3.3.1. Marsupial Records within the Context of All Species

**Figure 11:**
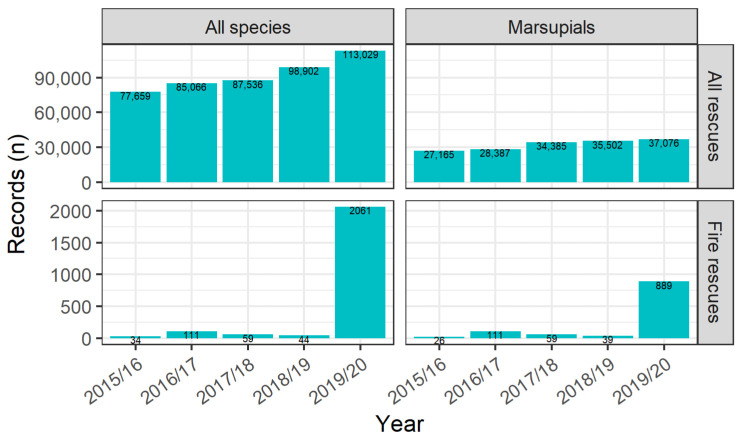
Wildlife rescue records in NSW between 2015/16 and 2019/20 for all species and for marsupials alone. Data sourced from NSW Wildlife Rehabilitation dashboard [29].

The number of rescue records reported to NPWS for all wildlife due to any cause increased year-on-year from 77,659 in 2015/16 to 98,902 in 2018/19, and 113,029 in 2019/20. Correspondingly, rescue records for marsupials increased annually from 27,165 in 2015/16 to 35,502 in 2018/29 and 37,076 in 2019/20 (Figure 11; Table S1). The five most common causes of rescue for all wildlife species from 2015/16 to 2019/20, excluding unknown causes, were motor vehicle collisions (24%), being in an unsuitable environment (14%), abandoned/orphaned (10%), other collisions (6%) and dependant on parent taken into care (6%) [29]. For marsupials, the most common causes for rescue from 2015/16 to 2019/20, excluding unknown causes, were motor vehicle collisions (41%), abandoned/orphaned (11%), being in an unsuitable environment (10%), dependant on parent taken into care (8%) and dog attacks (4%) [29]. In 2015/16, there were 5774 wildlife rehabilitation volunteers in NSW; this number declined to 5275 in 2016/17, gradually increased to 5602 in 2018/19 and increased to 6698 in 2019/20 [42]. There was no significant relationship between the number of rehabilitation volunteers and the total yearly wildlife rescues (F_1,18_ = 0.12, *p* = 0.736).

#### 3.3.2. Fire Records within the Context of All NSW Rescue Records

For 2015/16–2018/19, “Event—Fire” was the cause for 0.1% of all wildlife rescue records (*n* = 248 of 349,163), increasing to 1.8% in 2019/20 (*n* = 2061 of 113,029). When looking only at marsupial species, fire was the reported cause in 0.2% of all marsupial rescues for 2015/16–2018/19 (*n* = 235 of 125,439), increasing to 2.4% in 2019/20 (*n* = 889 of 37,076). Although this was an increase, fire still represented a small proportion of the total marsupial rescues in 2019/20. Fires resulted in 0.2% of mammal (*n* = 220 of 130,050), 0.01% of bird (*n* = 19 of 177,855), 0.02% of reptile (*n* = 9 of 40,597) and 0.0% of amphibian (*n* = 0 of 562) rescues from 2015/16 to 2018/19 and 4.1% of mammal (*n* = 1971 of 48,659), 0.6% of bird (*n* = 77 of 12,359), 0.1% of reptile (*n* = 13 of 10,477) and 0.0% of amphibian rescues (*n* = 0 of 203) in 2019/20 [29]. 

### 3.4. Comparison with Kangaroo Island 2019/20 Bushfires

#### 3.4.1. Frequency of Rescues and Triage Outcomes from Kangaroo Island Wildlife Park Hospital

There were 618 records of marsupials rescued due to the 2019/20 fires on Kangaroo Island and triaged at the Kangaroo Island Wildlife Park Hospital. The species most represented was the koala (84% of records), with relatively few wallabies, possums and kangaroos, and 22 listed as unknown species (Table 4). These are unlikely to be comprehensive records of the numbers of marsupials rescued due to difficulties in recording and collating data during the emergency response. The most common triage outcome for koalas was hospitalisation, while the most common outcome for other marsupials was euthanasia. Koalas were the only species with any released individuals reported, totalling 136 recorded releases. Looking specifically at wildlife rescued with a diagnosis of “burns”, most koalas were hospitalised (*n* = 170 of 211) and all other species were euthanised apart from one kangaroo (*n* = 35 of 36; Table S9). Most marsupial rescues on Kangaroo Island with a date recorded occurred during January 2020 (*n* = 239), after fires ignited on the north and north-eastern coasts on 20 December 2019 and in Flinders Chase National Park on 30 December 2019, before all fire zones were declared safe on 7 February 2020 [43].

#### 3.4.2. Pre-Fire Densities

The two macropod species were more abundant than other marsupial species on Kangaroo Island prior to 2019/20 (Table 5), highlighting the over-representation of koalas in the triage statistics relative to their baseline population density. Following the bushfires, DEW estimated that approximately 8500 koalas remained, based on the assumption that most koalas within the area impacted by the bushfires perished [44].

## 4. Discussion

Our study of the rescue and rehabilitation of fire-affected marsupials following the 2019/20 bushfire season represents the first detailed, multi-species account of the numbers of fire-affected marsupials rescued, treated and released across geographically extensive fire-affected regions, namely within the state of NSW and Kangaroo Island. The key, broad outcomes were as follows: (i) relatively few marsupials were reported to wildlife rehabilitation organisations, despite the estimates of large numbers of animals occurring within the bushfire zones [4]; (ii) there was a bias in the reporting of different marsupial species relative to pre-fire wildlife rehabilitation reporting rates and estimated baseline densities; (iii) rehabilitation outcomes varied significantly between species, from triage through to release; (iv) injury type predicted the likelihood of survival and subsequent release; and (v) the numbers of marsupials rescued following fires is only a small fraction of the number that enter rehabilitation from other misadventures in any given year. We discuss these outcomes further below.

### 4.1. Relatively Few Marsupials Were Rescued following 2019/20 Bushfires

Relatively few marsupials were reported to rehabilitation organisations despite an estimate of 3 billion animals being directly or indirectly impacted in fire zones across Australia [4]. It was estimated that 46.8 million marsupials were in the path of the 2019/20 fires in the state of NSW [4], yet only 889 marsupials in NSW were reported in need of rescue or assistance. Of these, 655 were rescued and 28% (*n* = 183) were subsequently released into the wild. These low reporting rates are undoubtedly the result of a combination of inter-related factors. The larger scale and greater intensity of the 2019/20 fires [45,46] likely prevented marsupials from evading the fire front or reaching unburnt refuges in cases where shelter was insufficient [31,47,48]. Most animals that attempted to shelter in place probably perished with the high fire intensity [49]. This hypothesis is supported by our results showing that a greater proportion of marsupials were reported dead on arrival of a rescuer during 2019/20 (16%), compared with previous fire events (1%). Additionally, the risk to human safety, and a likely lack of resources, delayed access to firegrounds to facilitate wildlife rescue [23]. This is supported by peak rescues occurring 6–8 weeks after fire ignition in NSW. Given the severity of the fires, it is likely that many animals that survived the immediate fire may have perished before rescue or moved to adjacent areas with more resources. The fact that most rescues of fire-affected marsupials were around the edge of the fire zone further suggests that access to firegrounds was difficult. It also probably reflects the increased visibility of animals along these interfaces, and a greater ability of animals to reach the edge of the fire zone and survive if close enough to begin with. Conversely, as there is considerable variation in fire intensity both geographically and temporally, animals situated within the intense fire zone were likely incinerated or dependent on fire response personnel for rescue. Despite animals needing rescue immediately post-fire, it took time before appropriate personnel could begin systematic searches. As detailed by Cristescu and Frere [50], a wildlife search team found koalas dying from starvation, dehydration, smoke inhalation and other hazards weeks after fires had passed. Similarly, koalas were rescued on Kangaroo Island over a span of 10 weeks [15]. 

The records may also be an under-estimate of the animals that were encountered in the field at the time of the fires. Some fires in northern NSW commenced before November and would not be reflected in our analyses, but it is unlikely that this is a large number, given the consistently low numbers reported from all other fires. It is not known to what extent all attempted rescues, especially euthanasia in the field or at over-run triage stations, were captured in recording systems during the unprecedented scale of the disaster. Nonetheless, it is likely that any animals that were in care for an extended period would be captured in wildlife rehabilitation statistics as part of statutory annual reporting, highlighting the low numbers of animals that survived to be rescued and then successfully rehabilitated following the 2019/20 bushfires. 

### 4.2. Some Marsupial Species Were Disproportionately Represented in 2019/20 Bushfire Rescue Statistics

We hypothesised that differing life-history strategies would contribute to differing rescue rates of marsupial species, independent of their relative pre-fire abundance within the ecosystem. This was clearly the case, with some species (most notably koalas) disproportionately represented in 2019/20 fire rescue statistics relative to estimates of their baseline population density (after van Eeden et al. [4]) and pre-fire wildlife rehabilitation reporting rates. 

In NSW, koalas represented 23% of 2019/20 bushfire marsupial records, compared with 11% of records during previous bushfire events, and 6% across all rescue types. Contrastingly, possums and gliders were under-represented during 2019/20, accounting for just 18% of the marsupial records in NSW, compared with their baseline pre-fire report rate (43%) and their reports during previous fire events (42%). The higher-than-expected number of koalas reported in NSW may be related to their patchy distribution [51], with the fire zones coinciding with some extant population strongholds in NSW [38]. However, it is unlikely that the bias is accounted for by this factor alone. There was no clear trend between baseline population density and 2019/20 records in each of the four major bioregions where fires occurred in NSW. For example, koalas were the most common marsupial species rescued on the NSW North Coast during 2019/20, despite estimated population densities that were several orders of magnitude lower than both possums and macropods [4,38]. Possums had the lowest reporting rate of all three groups on the NSW North Coast and a lower-than-expected number of records in the Sydney Basin (given their high pre-fire report rates and relatively high population density). Across these four bioregions, macropods were reported 1.2-fold more often than possums due to fires in 2015–19, but this rose to a 2.8-fold difference during the 2019/20 fires. These species biases were not as evident in the 2015–19 fire events at both the state (NSW) and bioregion level, further supporting the assertion that the severity and scale of the 2019/20 bushfires reduced the efficacy of fire avoidance behaviours for (at least some) wildlife [49]. 

This bias towards koalas was also observed in the state of Victoria, where most animals triaged at Zoos Victoria were koalas (75%) [13]. Koalas were also rescued in greater numbers than macropods on Kangaroo Island, representing 81% of wildlife triaged at Kangaroo Island Wildlife Park, a trend at odds with their estimated pre-fire densities. However, koalas were in the highest densities on the western half of the island where most of the forest burnt [52], whereas kangaroo and wallaby populations were at greater densities on the eastern side of the island where there was less fire activity. This could explain some of the disparity, but spatial variability in density alone cannot account for the significant disparity in reported rescue rates between the species. 

The reasons for these biases in marsupial reports during 2019/20 are not entirely clear. They likely reflect a combination of different life-history traits (as hypothesised) and practical decisions related to the capacity to rescue different species and differing prioritisation of species. There are clear differences in life-history traits between koalas, possums and kangaroos. These differences may affect any or all of the following: their behavioural response to fire, their capacity to evade fire and subsequent likelihood of initial survival, the extent and type of injuries sustained, and their behavioural response to people which may impact their capacity to be detected and/or rescued post-fire [53,54]. 

Koalas may have been more predisposed to injury due to their arboreal nature and minimal use of protective shelter, or koalas may have initially been protected in tree crowns and then injured when descending into firegrounds [55]. On Kangaroo Island, there were anecdotal reports of koalas sheltering in treetops where they escaped the worst of the fires, whereas kangaroos and wallabies could not outrun the fires [56]. However, the arboreal nature of koalas alone cannot account for the differential reporting rates. Possums are also arboreal yet were significantly under-represented in the 2019/20 bushfire records. The use of tree hollows or nests made of vegetation may have predisposed possums to injury or death within their shelters [57,58], and their nocturnal habits may have minimised their opportunity to be detected during daylight searches by rescuers, especially if they found protective shelter in deep tree hollows [59]. Injured koalas, on the other hand, may have been more visible to rescuers during the day than possums, especially in places where canopy foliage had been burnt away (although Cristescu and Frere [50] still found that koalas were difficult to locate without the aid of detection dogs and drones). 

An alternative, or additional, explanation may relate to human behaviour. The threatened status of koalas in NSW at the time of the fires [60], and their charismatic appeal internationally [61], may have created a bias towards targeted search and rescue of koalas. However, conservation status alone could not explain the disparity in rescue rates between koalas and other marsupials. Koalas on Kangaroo Island were considered overabundant and were actively being managed to reduce their density at the time of the fires [62], yet the bias towards koalas occurred in both locations. As described by Wilks [63], the perception of koalas as a unique species under threat and in need of conservation has extended to the koalas on Kangaroo Island even though they are “neither rare nor vulnerable and are of equivocal conservation value” (pg 1). Koalas also have the advantage of being easier for non-skilled rescuers to capture in comparison to large kangaroos or more cryptic species [64]. A recent study of the numbers of koalas rescued in NSW from the 2019/20 fires found that, along with fire-affected koalas, there was a spike in the number of healthy koalas being presented to koala rehabilitation groups [14]. This increased rescue of healthy koalas suggests that the community was hypervigilant towards koala health during the time of the 2019/20 bushfires [14].

### 4.3. Rescue and Rehabilitation Outcomes Varied between Species

We hypothesised that survival outcomes would vary between species. For the species rescued in sufficient numbers for analysis (macropods, koalas and possums), survival outcomes were significantly lower for macropods than other species. However, macropod release rates were low irrespective of the reason for entry into care (Table S2), with only 9.5% of macropods that entered care for any reason in the periods 2015/16–2018/19 and 2019/20 being subsequently released. In the 2019/20 fires, adult macropods also had a significantly lower probability of release than juveniles. This may be explained by the higher propensity for adult macropods to experience capture myopathy [65] and the difficulty capturing adult macropods without the aid of a specialist darter, unless they are largely incapacitated (i.e., moribund). The capacity to adequately house macropods for rehabilitation may also limit the capacity to treat these large animals. These factors may predispose them to higher death rates during or after capture, due to the stress of capture and/or the more advanced state of their incapacitation at the time of rescue, and the practicality of managing their rehabilitation may influence triage decisions. 

The likelihood of successful release of koalas during the 2019/20 bushfires in NSW was 47%, which is not dissimilar to the reports from Victoria and Kangaroo Island. Zoos Victoria had a high release rate of 42% within 24 hours of triage; 20% of animals were euthanised at triage, and 35% required ongoing treatment [13]. Of 239 koalas with triage records, death or euthanasia was recorded for 46%, and 54% were released [15]. The higher rates of survival to release for koalas and possums rescued following fires in NSW similarly mirror their higher release rates irrespective of the reason for rescue. 

We found that koalas in NSW had a longer length of stay (LOS) in rehabilitation than macropods or possums before euthanasia or death occurred. Most macropod and possum deaths and cases of euthanasia occurred within 24 hours of rescue, whereas koalas had a mean LOS of 27 days before death or euthanasia. This additional time in a potentially stressful environment [66] until death could present a welfare concern and warrants further investigation. There may be many reasons why koalas more frequently entered rehabilitation (e.g., their threatened status in NSW, or koalas generally being more amenable to treatment than macropods, for instance), and these should be explored through qualitative surveys or focus groups. Moreover, recent studies of triage assessments for fire-affected koalas on Kangaroo Island [15] and fire-affected koalas rehabilitated and released in Victoria [13] and their associated outcomes will help to inform triage decision-making in the future.

### 4.4. Predictors of Survival to Release

Injury type was consistently a predictor for the probability of release for koalas, macropods and possums, with malnourished/moribund animals and those suffering from traumatic injuries having relatively low probabilities of release and abandoned or orphaned animals having higher probabilities of release. This was consistent with our hypothesis and the findings in our systematic review of factors affecting wildlife survival during rehabilitation [28]. 

The 2019/20 bushfire injury types were mostly traumatic, including the presence of burns, as would be expected. In NSW, injury was recorded more often than burns under the trauma category in 2019/20 compared with the preceding four years for fire-affected animals, suggesting there may have been inconsistencies in the way injuries and burns were recorded between years or rehabilitation groups, or an indication that injury was more common than burns in 2019/20. Our results are consistent with the analysis of koala triage records on Kangaroo Island by Dunstan et al. [15], who found that death or euthanasia of koalas was related to higher burn score, severity, and number of regions burnt and poor body condition and hydration level [15]. There is also evidence that smoke inhalation caused wildlife mortality at sites more than 20 km from the fire zone [67]; hence, there may be broader health impacts of the fires on wildlife that have not yet been recognised or quantified. 

There was no effect of age or sex on the probability of release in most cases (although juvenile macropods were more likely to be released than adults, as discussed above). There were more adult koalas, macropods and possums reported due to fires in 2019/20 than juveniles, similar to fires in previous years. There were no sex biases in rescues of fire-affected koalas, macropods or possums in 2019/20, differing from fires in previous years where female koalas and possums were more commonly rescued than males. Comparatively, there were no age or sex biases in koalas rescued on Kangaroo Island in the 2019/20 fires [15].

We also hypothesised that survival outcomes would vary with fire severity, but fire severity was not a significant predictor of survival overall for koalas, macropods or possums rescued in NSW. The one exception was that macropods rescued outside the fire zone had a higher probability of release than those found within, again highlighting the importance of proximity to unburnt refuges for survival in high-severity fires [31,68]. A systematic review of animal mortality during fire found that fires of higher severity caused greater mortality rates than fires of low severity [69]. A study of the impacts of the 2019/20 bushfires on koala populations found that koala survival was five times more likely in areas of unburnt or partially burnt canopy compared to fully burnt canopies [70]. Lindenmayer et al. [71] found that the southern greater glider (*Petauroides volans*) responded negatively to fire severity at the site level and to the amount of burnt forest in the surrounding landscape, and arboreal marsupials were more abundant at unburnt sites with greater availability of hollow-bearing trees. Our study could not account for all mortalities across fire severity zones, as there was no consistent reporting of carcasses found, which likely explains why our findings do not align with these other studies. Similarly, Dunstan et al. [15] found there was no relationship between the presence of burns and the rescue location of koalas in terms of fireground, fire severity or vegetation type for koalas on Kangaroo Island, but very few rescues were within high-severity firegrounds [15]. Together, these findings suggest that while fire severity may be a significant predictor of survival in situ in the immediate aftermath of the fire, it is not an important predictor of survival during rehabilitation ex situ. 

### 4.5. Fires Only Account for a Small Fraction of Wildlife Rescues

The 2019/20 fires placed wildlife rescuers and rehabilitators on the front page of news reports internationally [72] and presented a substantial spike in pressure on volunteers and veterinary hospital resources when communities were focused on fighting to protect human life, pets and livelihoods [73]. But, in reality, fires only account for a very small proportion of the wildlife in need of assistance. Even in a fire season as extraordinary as 2019/20, fires only accounted for 1.8% of all wildlife entering care (up from 0.1% in the preceding years) and 2.4% for marsupials (up from 0.2%). This highlights the need for additional focus on “peacetime” wildlife rescue and rehabilitation. Although less sensational, the plight of wildlife impacted by day-to-day interactions with people, their vehicles and their pets should be equally newsworthy.

### 4.6. Limitations

In interpreting our results, it is important to acknowledge the unbalanced and potentially biased nature of the wildlife rehabilitation database utilised. The accuracy of records relies on volunteers inputting their rescue data correctly and completely and updating the record with details of fate. There were many cases of missing data and potential for inconsistencies in the classifications attributed to cases; for example, the cause for rescue, injury type and age definitions could be subjective between wildlife rehabilitation groups. Also, NPWS does not receive data from veterinary hospitals that receive animals from the public that are subsequently euthanised and do not enter the rehabilitation sector pathway [74]. Fortunately, it appears that only a small proportion of fire-affected animals in the summer of 2019/20 had an unclassified “cause for rescue” reported (3.7% of the reviewed unclassified records for eastern grey kangaroos, brushtail possums and ringtail possums from WIRES during this time were found to be fire-related). Rehabilitation records can only account for animals that were found (dead or alive) in areas where humans were present or searching, so they are an inherently incomprehensive account of all fire-affected animals in the fire zone and surrounding areas. The distribution of wildlife rescue efforts in NSW was not even across all the fire zones. It was also variable as to when people could access the fireground due to safety, ongoing fire, road closures and a focus on protecting property and human lives. Yet, in the absence of systematic on-ground surveys before, during and after bushfire events, wildlife rehabilitation records provide a valuable source of information on trends in wildlife rescue and survival. Our analyses have focused on the more common species that were encountered in the aftermath of the 2019/20 bushfires. Fire-affected bettongs, potoroos, bandicoots and small dasyurids were reported in very low numbers across all years, suggesting that either very few survived fires (as has been previously reported; [57,58]), very few were injured, or they are difficult to find post-fire.

### 4.7. Management Implications

In some ways, it seems that wildlife rescue and rehabilitation during and immediately after the 2019/20 bushfires were amplified in the media, resulting in a disconnect between the public’s perception of wildlife rescue and the reality of the situation. The national and international community donated millions of dollars to wildlife rescue in Australia; however, very few animals survived to benefit from financial support [14,75,76]. The broader community clearly values wildlife rescue services but is not fully aware of how such organisations are funded or operated [77,78]. Increased awareness and continued community education are important to mediate expectations for wildlife rehabilitation after extreme events and generate support for the day-to-day and emergency operations of these largely volunteer-based organisations.

Trained wildlife emergency response teams are an important component of emergency response, especially in the period post-fire, as shown by the large numbers of animals found needing treatment or euthanasia 6–8 weeks after fire ignition in our study. In a positive step forward, the Wildlife Emergency Response Team’s capacity to respond to injured wildlife on the fireground has been built through training, workshops and the provision of fireground PPE to the veterinary and wildlife rehabilitation sectors in NSW ([79]; pers. comm. S. Grootemaat [NPWS] 2024). Additionally, firefighters in NSW now receive wildlife first response training, increasing their capacity in wildlife response [22,64]. Further examples of wildlife rescue teams include the detection dogs and drones used by Cristescu et al. [80]; the macropod specialists and darters, wildlife veterinarians and vet nurses sent by WIRES (pers. comm. Zoe Harrison [WIRES] 2021); and the Australian Veterinary Emergency Response Team (AVERT) [81]. The NSW Government has also implemented veterinary professional development training in wildlife treatment and care through the NSW Koala Strategy. NSW DPIE has recently updated the Rehabilitation Training Standards for possums, gliders [82] and koalas [83]. Initial care and treatment guidelines have also been developed for a range of marsupial species to guide licensed wildlife rehabilitators on assessment and first aid principles. There is a need for increased numbers of trained macropod darters and shooters to support these post-fire rescue efforts and minimise wildlife suffering [23]. There is also a lack of consistency in the reporting of wildlife rescue and rehabilitation between jurisdictions, which hampers attempts to learn from these disasters. New technologies should be implemented to improve the ease and quality of reporting wildlife encounters (including mortalities and GPS rescue location identification) in the field and to easily track animals through the rescue–rehabilitation–release process. Greater collaboration between jurisdictions in developing standards for reporting on emergency wildlife response is likely to enhance welfare outcomes.

Individual animal welfare should always be prioritised no matter the species, and all efforts be made to find affected animals as efficiently as possible. The findings of this study, and those of Parrott et al. [13] and Dunstan et al. [15], should be used to refine triage decisions so that animals with a reasonable prognosis of survival can be prioritised for rehabilitation and to identify areas where more research may be necessary to develop improved standards of care. The poor release rates for macropods rescued across the board also highlight the need for more research on improving rehabilitation outcomes for rescued macropods. While we have focused on the broad trends relating to marsupial rescue and rehabilitation post-fire, the fine-scale detail provided in the body of this paper (and Supplementary Materials) will aid wildlife rehabilitators, governmental bodies and other organisations in their preparedness for future natural disasters on a species-by-species basis, and at a regional level. 

The post-fire environment poses short-term and long-term risks to surviving or released wildlife, and mitigation and support strategies should be areas for future research, particularly as wildfires are likely to increase in intensity and scale in the future [84]. Funding of management actions to address the challenges posed to animals in the post-fire landscape, such as predation, starvation and dehydration, in the short and long term is also crucial [21,69,76], along with ongoing research and monitoring to evaluate the success of such efforts, and the best approach for future fire events [85]. The plight of wildlife in the aftermath of a fire must also be viewed within the context of the myriad of threats they face. 

We found that 28% of rescued fire-affected marsupials were released from rehabilitation in NSW in 2019/20; however, we do not know what the post-release survival rate was. Few studies have monitored post-release survival of rehabilitated wildlife, and even fewer have included an experimental design or control group [28]. A study of hand-raised brushtail possums found that 45% of releases were unsuccessful in the first four weeks [86], yet, from a long-term rehabilitation dataset, we know that rehabilitated koalas are capable of surviving in the wild long-term [87]. A study of koalas rehabilitated following fire found that the annual post-release survival rate was 58% compared with 67% for uninjured koalas, and predation by dogs was the leading cause of mortality in both cohorts [88]. Other studies found that 36 released rehabilitated koalas had an overall survival rate of 58.5%, and a critical threshold for mortality at two weeks post-release [89], and that rehabilitated koalas can be released into burnt habitats (with adequate nutrition) without their health being compromised [90]. Factors affecting post-release survival are context- and species-specific [28], and further studies are therefore needed to determine the post-release survival of fire-affected wildlife species. The release environment quality is likely to be a significant consideration in the release of rehabilitated fire-affected wildlife [91]. The availability of suitable release locations within a short timeframe can be a challenge, particularly as unburnt habitats are likely to be refuges for fire survivors and already be at capacity. Research on the suitability and feasibility of translocation of rehabilitated wildlife is needed in situations where suitable release locations are limited [28], as keeping animals in care for longer periods will likely increase levels of humanisation, which has been linked with poor post-release outcomes [86].

## 5. Conclusions

The 2019/20 bushfires in south-eastern Australia had widespread, devastating effects on communities and the environment [5,18,45,92,93]. The impacts on wildlife were particularly emotive to the public, described at the time as “difficult to fathom” and “overwhelming and distressing” [94]. However, it is impossible to quantify the true impact of the 2019/20 bushfires on wildlife due to a lack of data on pre- and post-fire population densities and short- and long-term mortality rates resulting from the fires. As stated in an Australian Senate report, “there is a clear need for better data collection across the board, highlighted by the fact there was no baseline data going into the 2019–20 bushfire season, and that it is still hard to quantify the total impact of the 2019/20 bushfires on both flora and fauna” [94] (p. 102).

Our study presents the first account of the numbers of marsupials rescued, treated and released in NSW, which places the existing accounts of koala rescues during the 2019/20 bushfires [13,14,15] into a broader context. We found that koalas were rescued more frequently than macropods and possums, and at a higher rate than would be expected based on their underlying densities. The reasons for this bias should be further investigated. The scale and severity of the 2019/20 fires meant that very few animals were rescued, and delays in ensuring safe access to firegrounds meant that some animals suffered for several weeks prior to rescue. Given the low numbers of marsupials rescued and released after the fires, one could question the worth of rehabilitation. Yet, while it is difficult to quantify the contribution of wildlife rehabilitation to the conservation of species, volunteer wildlife rehabilitators provide an essential component of the wildlife response post-disaster. There is a clear need for stakeholders to work with them to develop efficient, well-equipped and systematic wildlife rescue responses and evidence-based triage decisions after environmental disasters to improve individual animal welfare outcomes. Stories of wildlife rehabilitation, particularly of koalas, also successfully draw the world’s attention to the plight of Australia’s wildlife in an age of climate change and species extinction [95].

## Figures and Tables

**Table 1:** Frequency of fates of marsupials reported to NPWS that were rescued due to “Event—Fire” in 2019/20.

Marsupial Group	Advice/Sighting/Transferred	Dead on Arrival	Euthanised/ Died in Care	Released/ Relocated	In Care	Unknown	Total
Macropods	51	108	225	37	21	16	458
Koalas	20	20	70	63	18	13	204
Possums and gliders	7	13	61	68	9	4	162
Wombats	7	4	8	2	21	1	43
Dasyurids	1	0	1	11	0	0	13
Bandicoots	0	0	4	2	0	0	6
Unknown	2	0	0	0	0	0	2
Bettongs and potoroos	1	0	0	0	0	0	1
Total	89	145	369	183	69	34	889

**Table 2:** Linear regression model output for significant predictors of length of stay for fire-affected macropods, koalas, and possums and gliders that were released/relocated or euthanised/died in care.

Released/Relocated		
**Macropods (*n* = 37)**	Injury type	R^2^ = 0.55, F_3,25_ = 10.2, *p* < 0.001, Figure 7a
	Age	R^2^ = 0.15, F_1,27_ = 4.6, *p* = 0.041
**Koalas** **(*n* = 63)**	Fire severity	R^2^ = 0.14, F_4,56_ = 3.83, *p* = 0.015, Figure 7b
	Injury type	R^2^ = 0.17, F_4,51_ = 2.5, *p* = 0.05, Figure 7c
	Age	R^2^ = 0.19, F_1,60_ = 14.1, *p* < 0.001
	Sex	R^2^ = 0.07, F_1,60_ = 4.3, *p* = 0.04
	Fire site	R^2^ = 0.13, F_8,52_ = 2.2, *p* = 0.047
**Possums** **(*n* = 68)**	Injury type	R^2^ = 0.22, F_4,48_ = 3.3, *p* = 0.018, Figure 7d
	Fire site	R^2^ = 0.60, F_23,17_ = 3.7, *p* = 0.004
**Euthanised/Died in Care**		
**Macropods (*n* = 333)**	Injury type	R^2^ = 0.10, F_3,194_ = 7.2, *p* < 0.001, Figure 8a
	Age	R^2^ = 0.06, F_1,181_ = 11.8, *p* < 0.001, Figure 8b
	Fire site	R^2^ = 0.28, F_31,161_ = 3.5, *p* < 0.001
**Koalas** **(*n* = 90)**	Fire site	R^2^ = 0.24, F_13,44_ = 2.4, *p* = 0.017
**Possums** **(*n* = 74)**	N/A	

**Table 3:** Proportion of fire-affected marsupial reports per fire severity rating (%) and probability of release (mean ± s.e.) after rescue from each zone for koalas, macropods, and possums and gliders.

Marsupial Group	No Severity Data (%; Mean ± s.e.)	Nonburnt (%; Mean ± s.e)	Medium (%; Mean ± s.e)	High (%; Mean ± s.e)	Extreme (%; Mean ± s.e)	Total (n; Mean ± s.e)	No Spatial Data (n)
**Koalas**	10 (0.4 ± 0.1)	46 (0.4 ± 0.1)	9 (0.3 ± 0.1)	15 (0.4 ± 0.1)	20 (0.7 ± 0.1)	128 (0.5 ± 0.1)	34
**Macropods**	20 (0.1 ± 0)	36 (0.1 ± 0.0)	15 (0.0 ± 0)	15 (0.1 ± 0.1)	14 (0.1 ± 0)	232 (0.1 ± 0.0)	61
**Possums**	5 (0.4 ± 0.2)	64 (0.5 ± 0.1)	10 (0.3 ± 0.1)	12 (0.1 ± 0.1)	10 (0.6 ± 0.1)	110 (0.5 ± 0.1)	30

**Table 4:** Triage decision (n) and final fate per species brought to the Kangaroo Island Wildlife Park Hospital during the 2019–2020 bushfires.

Species	Records (n)	Triage Decision	Triage (n)/Fate
Koala	517	Hospitalise	*n* = 225 (87 = euthanised/died, 86 = released, 51 = not recorded, 1 = captivity)
		Euthanise	*n* = 18
		Releasable	*n* = 67 (6 = died/euthanised, 50 = released, 11 = not recorded)
		Dead on arrival	*n* = 3
		Not recorded	*n* = 204
Wallaby	36	Euthanise	*n* = 14
		Not recorded	*n* = 22
Possum	28	Euthanise	*n* = 21
		Not recorded	*n* = 7
Kangaroo	15	Euthanise	*n* = 7
		Hospitalise	*n* = 1 (1 = permanent captivity)
		Not recorded	*n* = 7
Unknown	22	Not recorded	*n* = 22
Total	618		

**Table 5:** Population density and size estimates for tammar wallabies, western grey kangaroos and koalas on Kangaroo Island, SA, prior to the 2019/20 wildfires.

Species	Population Density Estimate	Population Estimate	Number Rescued
Tammar wallaby	0.16 per ha occupy 439,800 ha	70,588 ^#^ [39]	36
Western grey kangaroo	0.15 per Ha [39] occupy 439,800 ha	65,882 [39]	15
Koala	0.11 per Ha (across entire island) *	48,506 ± 5976 ** [40]	517
Brushtail possum	0.1 per ha occupy 439,800 ha [4]	43,980	28
Total			618

^#^ Reference [39] gives a total population size of 87,043 for tammar wallabies, but given a density of 0.1605/ha and a distribution across the entire island, this totals 70,588; * 1.20 ± 0.17 koalas per ha (up to 4.19/ha) [0.012 ± 0.0017/km^2^] in high-quality habitats [40]; ** 25,146 ± 2646 in native vegetation plus 23,360 ± 3330 in commercial blue gum plantations [40].

## Data Availability

Restrictions apply to the availability of these data. Data were obtained from NSW National Parks and Wildlife Service and Zoos SA and should be requested from them directly.

## Supplementary Materials

The following supporting information can be downloaded at https://www.mdpi.com/article/10.3390/ani14071019/s1: Table S1: Animal rescue records in NSW between 2015/16 and 2019/20 for all rescue types, with “Event—Fire” records presented in brackets. Table S2: Records, admissions and release rate for all marsupial rescues (including fire) and marsupial “Event—Fire” rescues (presented in brackets) for 2015/16–2018/19 and 2019/20. Table S3: Frequency of fates of marsupials reported to NPWS that were rescued due to “Event—Fire” in 2019/20 and 2015/16 to 2018/19 and the release rate. Table S4: Number and type of injury per marsupial group rescued due to fire in 2019/20 and 2015/16–2018/19 and percentage represented by each injury type overall (injury types presented from most to least common by %). Table S5: Summary of general linear model output for potential predictors of release for macropods, koalas, and possums and gliders (bold text indicates significant *p*-values < 0.05). Separate models were conducted for each explanatory variable within each marsupial group due to differing sample sizes and availability of data. Fates compared were euthanised/died in care vs. released/relocated and exclude those found dead on arrival of the rescuer. Table S6: Model output from Poisson regression model to assess any sex or age biases in rescues for all causes and fire-related causes, and Pearson’s chi-squared test to assess whether there was a shift in the bias between pre-megafire years (2015/16–2018/19) and megafire years (2019/20) for macropods, koalas, possums and gliders, and wombats. Cells containing significant *p*-values have been shaded. Table S7: Number of koalas, macropods, and possums and gliders rescued due to fires in 2019/20 split into weeks since fire ignition (total *n* = 591 with accurate dates for both rescue and fire ignition and excluding records that were dead prior to rescuer arriving). Table S8: Summary of the model results for predictors of length of stay for macropods, koalas, and possums and gliders (bold text indicates significant *p*-values < 0.05). Table S9: Triage outcome for wildlife rescued with burn injuries via Kangaroo Island Wildlife Park Hospital. Figure S1: Map of the eastern coast of NSW, Australia, depicting the location of bushfire-related marsupial reports in 2019/20 in relation to the fire extent.
