# Supplementary material for: Trends in Rescue and Rehabilitation of Marsupials Surviving the Australian 2019–2020 Bushfires
Source: Animals (Basel). 2024 Mar 27;14(7):1019. doi: 10.3390/ani14071019 (PMC11011103; doi:10.3390/ani14071019)

Supplementary Files

Table S1. Animal rescue records in NSW between 2015/16 and 2019/20. Data sourced from NSW Wildlife Rehabilitation dashboard [1] and NSW Wildlife Rehabilitation 2019-20 annual report [2]

**Rescues reported to NPWS**	**2015/16**	**2016/17**	**2017/18**	**2018/19**	**2019/20**	**Total**
All rescue records						
all species (n)*	77,659	85,066	87,536	98,902	113,029	462,192
marsupials (n)	27,165	28,387	34,385	35,502	37,076	162,515
marsupials admitted (n)	17,590	17,624	21,157	22,031	20,487	98,889
'Event – Fire' records						
all species (n)*	34	111	59	44	2,061	2,309
marsupials (n)	26	111	59	39	889	1124
marsupials admitted (n)	20	98	47	34	672	871
Wildlife rehabilitation volunteers (n)	5,774	5,275	5,374	5,602	6,698	N/A

*Admissions for all species could not be calculated from the data supplied via the NSW wildlife rehabilitation dashboard

1. DPIE. NSW wildlife rehabilitation dashboard. Available online: https://www.environment.nsw.gov.au/topics/animals-and-plants/native-animals/rehabilitating-native-animals/wildlife-rehabilitation-reporting/wildlife-rehabilitation-data (accessed on 17 January 2022).
2. DPIE. *NSW wildlife rehabilitation annual report 2019-20*; NSW Department of Planning, Industry and Environment: Parramatta, NSW, 2021; pp. 1-44.

Table S2. Records, admissions and release rate for all marsupial rescues (including fire) and marsupial 'Event – Fire' rescues for 2015/16 - 2018/19 and 2019/20.

Fire-related Rescues						
	2015/16 - 2018/19			2019/2020		
**Marsupial Groups**	**Records (n)**	**Admissions (n)**	**Releases* (%)**	**Records (n)**	**Admissions (n)**	**Releases* (%)**
Bandicoots	3	3	0	6	6	33.3
Bettongs and potoroos	0	0	NA	1	1	0
Dasyurids	3	3	66.7	13	12	91.7
Koalas	27	15	66.7	204	164	38.4
Macropods	101	85	5.9	458	299	12.4
Kangaroos	*80*	*69*	*4.3*	*341*	*209*	*10.5*
Kangaroo/wallaby	*2*	*2*	*0*	*16*	*9*	*0*
Wallabies	*19*	*14*	*14.3*	*101*	*81*	*18.5*
Possums and gliders	98	88	36.4	162	142	47.9
Wombats	2	1	0	43	32	6.3
Unknown	1	1	0	2	1	0
**Total**	**235**	**196**	**25.0**	**889**	**657**	**27.9**
**All Causes for Rescue**						
	**2015/16 - 2018/19**			**2019/2020**		
**Marsupial Groups**	**Records (n)**	**Admissions (n)**	**Releases* (%)**	**Records (n)**	**Admissions (n)**	**Releases* (%)**
Bandicoots	1,716	1,156	29.0	384	259	29.3
Bettongs and potoroos	27	25	28.0	17	16	62.5
Dasyurids	647	452	37.6	177	121	37.2
Koalas	7,831	3,194	37.9	2,979	1,177	41.5
Macropods	44,690	29,702	9.5	13,086	6,620	9.5
Kangaroos	*24,719*	*18,851*	*0.8*	*7703*	*3,809*	*0.7*
Kangaroo/wallaby	*12,268*	*5,368*	*30.0*	*3226*	*1,394*	*26.3*
Wallabies	*7,703*	*5,483*	*19.4*	*2157*	*1,417*	*16.8*
Possums and gliders	53,888	37,780	33.0	15,917	10,779	29.7
Wombats	6,058	3,211	13.7	1,986	896	7.0
Unknown	10,582	2,882	20.3	2,530	619	13.6
**Total**	**125,439**	**78,402**	**23.0**	**37,076**	**20,487**	**22.4**

*Released, relocated or reunited with parents of those admitted.

Table S3. Frequency of fates of marsupials reported to NPWS that were rescued due to 'Event – Fire' in 2019-2020 and 2015/16 to 2018/19 and the release rate.

2019-2020								
**Marsupial group**	**Advice/ Sighting/ Transferred**	**Dead on arrival**	**Died/ Euthanased in care**	**Released/ Relocated**	**In care/ Escaped from care**	**Unknown**	**Total**	**Released (%)***
Bandicoots	0	0	4	2	0	0	6	33.3
Bettongs and potoroos	1	0	0	0	0	0	1	-
Dasyurids	1	0	1	11	0	0	13	91.7
Koalas	20	20	70	63	20	11	204	38.4
Macropods	51	108	225	37	27	10	458	12.4
Kangaroo/wallaby	*7*	*0*	*7*	*0*	*0*	*2*	*16*	*0.0*
Kangaroos	*34*	*98*	*163*	*22*	*19*	*5*	*341*	*10.5*
Wallabies	*10*	*10*	*55*	*15*	*8*	*3*	*101*	*18.5*
Possums and gliders	7	13	61	68	12	1	162	47.9
Wombats	7	4	8	2	21	1	43	6.3
Unknown	2	0	0	0	0	0	2	-
**Total**	**89**	**145**	**369**	**183**	**80**	**23**	**889**	**27.9**
**Total (% of 889)**	**10.0**	**16.3**	**41.5**	**20.6**	**9.0**	**2.6**	**-**	
**2015/16 to 2018/19**								
**Marsupial group**	**Advice/ Sighting/ Transferred**	**Dead on arrival**	**Died/ Euthanased in care**	**Released/ Relocated**	**In care/ Escaped from care**	**Unknown**	**Total**	**Released (%)***
Bandicoots	0	0	3	0	0	0	3	0.0
Dasyurids	0	0	1	2	0	0	3	66.7
Koalas	12	0	4	10	1	0	27	66.7
Macropods	15	1	47	5	28	5	101	5.9

Kangaroo/wallaby	*0*	*0*	*1*	*0*	*0*	*1*	*2*	*0.0*
Kangaroos	*10*	*1*	*37*	*3*	*25*	*4*	*80*	*4.3*
Wallabies	*5*	*0*	*9*	*2*	*3*	*0*	*19*	*14.3*
Possums and gliders	8	2	44	32	10	2	98	36.4
Wombats	1	0	0	0	1	0	2	0.0
Unknown	0	0	0	0	1	0	1	0.0
**Total**	**36**	**3**	**99**	**49**	**41**	**7**	**235**	**25.0**
**Total (% of 235)**	**15.3**	**1.3**	**42.1**	**20.9**	**17.4**	**3.0**	**-**	

* Calculated as Released/relocated of those admitted for each marsupial group.

Table S4. Number and type of injury per marsupial group rescued due to fire in 2019/20 and 2015/16 – 2018/19 and percentage represented by each injury type overall (injury types presented from most to least common by %).

2019/20												
					Macropods							
**Injury type**	**Bandicoots**	**Bettongs and potoroos**	**Dasyurids**	**Koalas**	**Kangaroo / wallaby**	**Kangaroos**	**Wallabies**	**Possums and gliders**	**Wombats**	**Unknown**	**Total**	**Total (% of 889)**
Trauma	4	0	1	110	10	286	68	94	11	2	586	65.9
Injury	*2*	*0*	*1*	*17*	*10*	*216*	*44*	*56*	*6*	*0*	*352*	*39.6*
Burns	*2*	*0*	*0*	*93*	*0*	*66*	*24*	*33*	*4*	*2*	*224*	*25.2*
Respiratory difficulty	*0*	*0*	*0*	*0*	*0*	*4*	*0*	*5*	*1*	*0*	*10*	*1.1*
Abandoned/ orphaned	0	1	11	5	0	10	10	20	15	0	72	8.1
Other	2	0	1	19	1	16	6	20	4	0	69	7.8
Heat stress/ dehydrated	0	0	0	25	0	11	4	15	3	0	58	6.5
Malnourished/ moribund	0	0	0	26	1	7	5	7	6	0	52	5.8
Unknown/ unclassified	0	0	0	19	4	11	8	6	4	0	52	5.8
**Total**	**6**	**1**	**13**	**204**	**16**	**341**	**101**	**162**	**43**	**2**	**889**	-
**2015/16 – 2018/19**												
					Macropods							
**Injury type**	**Bandicoots**	**Bettongs and potoroos**	**Dasyurids**	**Koalas**	**Kangaroo/ wallaby**	**Kangaroos**	**Wallabies**	**Possums and gliders**	**Wombats**	**Unknown**	**Total (n)**	**Total (% of 235)**
Trauma	3	0	0	12	2	71	14	56	0	1	159	67.7
Burns	*0*	*0*	*0*	*8*	*1*	*63*	*9*	*18*	*0*	*0*	*99*	*42.1*
Injury	*3*	*0*	*0*	*4*	*1*	*8*	*5*	*36*	*0*	*1*	*58*	*24.7*

Respiratory difficulty	*0*	*0*	*0*	*0*	*0*	*0*	*0*	*2*	*0*	*0*	*2*	*0.9*
Other	0	0	0	3	0	2	1	12	0	0	18	7.7
Abandoned/ orphaned	0	0	3	0	0	3	2	9	0	0	17	7.2
Heat stress/ dehydrated	0	0	0	6	0	0	0	3	0	0	9	3.8
Malnourished/ moribund	0	0	0	0	0	0	2	4	0	0	6	2.6
Unknown/ unclassified	0	0	0	6	0	4	0	14	2	0	26	11.1
**Total**	**3**	**0**	**3**	**27**	**2**	**80**	**19**	**98**	**2**	**1**	**235**	-

Table S5. Summary of general linear model output for potential predictors of release for macropods, koalas, and possums and gliders (bold text indicates significant p-values < 0.05). Separate models were conducted for each explanatory variable within each marsupial group due to differing sample sizes and availability of data. Fates compared were euthanised/died in care vs released/relocated and excludes those found dead on arrival of the rescuer.

**Macropods**					
**Explanatory variable**	**Deviance explained (%)**	**n**	**LR χ^2**	**df**	**p-value**
Injury type	33.1	251	69.4	4	**< 0.001**
Age	2.1	236	4.3	1	**0.037**
Sex	0.1	212	0.2	1	0.631
Fire severity	1.4	150	3.6	4	0.460
Rescue within fire zone	2.1	231	2.9	1	0.090
Days since fire ignition when rescued	0.2	227	0.4	1	0.549
**Koalas**					
Injury type	7.9	124	13.5	4	**0.009**
Age	2.0	133	3.7	1	0.056
Sex	0.1	133	0.2	1	0.647
Fire severity	3.7	87	7.1	4	0.131
Rescue within fire zone	0.0	128	0.0	1	0.875
Days since fire ignition when rescued	0.3	127	0.5	1	0.486
**Possums and gliders**					
Injury type	13.7	125	23.8	4	**< 0.001**
Age	0.9	122	1.6	1	0.208
Sex	0.0	116	0.0	1	0.827
Fire severity	7.7	43	13.0	4	**0.011**
Rescue within fire zone	0.2	109	0.2	1	0.623
Days since fire ignition when rescued	3.6	98	6.0	1	**0.014**

Table S6. Model output from Poisson regression model to assess any sex or age biases in rescues for all causes and fire-related causes, and Pearson's chi-squared test to assess whether there was a shift in the bias between pre-mega fire years (2015/16 – 2018/19) and mega-fire years (2019/20), for macropods, koalas, possums and gliders, and wombats. Cells containing significant p-values have been shaded.

	**All causes: Overall bias?**	**All causes: Shift in bias before and during megafires?**	**Fire rescues: Overall bias?**	**Fire rescues: Shift in bias before and during megafires?**
	**Sex (Male vs Female)**			
**Macropods**	LR χ^2_1 = 186.79, $p <$ 0.001 i.e. M > F	χ^2_1 = 0, p = 1 i.e. no change	LR χ^2_1 = 2.19, p = 0.139 i.e. M = F	χ^2_1 = 1.46, p = 0.228 i.e. no change
**Koalas**	LR χ^2_1 = 21.71, $p <$ 0.001 i.e. M > F	χ^2_1 = 0, p = 1 i.e. no change	LR χ^2_1 = 0.25, p = 0.618 i.e. M = F	χ^2_1 = 6.27, p = 0.012 i.e. F > M in preceding years, F = M in 2019/20
**Possums and gliders**	LR χ^2_1 = 128.35, $p <$ 0.001 i.e. F > M	χ^2_1 = 0, p = 0.99 i.e. no change	LR χ^2_1 = 0.95, p = 0.329 i.e. M = F	χ^2_1 = 3.64, p = 0.056 i.e. no change*
	**Age (Juvenile vs Adult)**			
**Macropods**	LR χ^2_1 = 257.11, $p <$ 0.001 i.e. J > A	χ^2_1 = 0.44, p = 0.506 i.e. no change	LR χ^2_1 = 17.08, $p <$ 0.001 i.e. A > J	χ^2_1 = 0.87, p = 0.351 i.e. no change
**Koalas**	LR χ^2_1 = 2464.7, $p <$ 0.001 i.e. A > J	χ^2_1 = 0.04, p = 0.844 i.e. no change	LR χ^2_1 = 5.44, p = 0.020 i.e. A > J	χ^2_1 = 0.47, p = 0.494 i.e. no change
**Possums and gliders**	LR χ^2_1 = 1096.9, $p <$ 0.001 i.e. J > A	χ^2_1 = 0.01, p = 0.909 i.e. no change	LR χ^2_1 = 23.06, $p <$ 0.001 i.e. A > J	χ^2_1 = 0.24, p = 0.625 i.e. no change

*trend for slightly more males than females in 2019/20 than preceding years.

Table S7. Number of koalas, macropods, and possums and gliders rescued due to fires in 2019/20 split into weeks since fire ignition (total n = 591 with accurate dates for both rescue and fire ignition and excluding records that were dead prior to rescuer arriving).

Weeks since fire ignition	Koalas	Macropods	Possums and gliders	All marsupials
0 - 2 weeks	35	44	18	108
2 - 4 weeks	12	33	13	62
4 - 6 weeks	15	21	5	36
6 - 8 weeks	43	127	36	232
8 - 12 weeks	7	30	13	52
3 - 6 months	5	13	7	28
6 - 12 months	0	0	3	3

Table S8. Summary of the model results for predictors of length of stay for macropods, koalas and possums and gliders (bold text indicate significant p-values < 0.05).

Released/Relocated												
	**Macropods (n = 37)**				**Koalas (n = 63)**				**Possums and gliders (n = 68)**			
	**F**	**df, ddf**	**p-value**	**R^2**	**F**	**df, ddf**	**p-value**	**R^2**	**F**	**df, ddf**	**p-value**	**R^2**
**Injury type**	10.2	3,25	**< 0.001**	0.55	2.5	4,51	**0.051**	0.17	3.3	4,48	**0.018**	0.22
**Age**	4.6	1,27	**0.041**	0.15	14.1	1,60	**< 0.001**	0.19	0.1	1,49	0.707	0.00
**Sex**	2.8	1,27	0.109	0.09	4.3	1,60	**0.042**	0.07	0.2	1,47	0.625	0.00
**Fire severity**	2.7	4,12	0.079	0.30	3.38	4,56	**0.015**	0.14	0.13	4,36	0.969	0.00
**Rescue within fire zone**	0.2	1,15	0.698	0.01	0.0	1,59	0.925	0.00	2.9	1,38	0.096	0.07
**Days since fire ignition when rescued**	0.6	1,15	0.448	0.00	0.53	1,59	0.468	0.00	1.0	1,39	0.329	0.00
**Died/Euthanased in care**												
	**Macropods (n = 333)**				**Koalas (n = 90)**				**Possums and gliders (n = 74)**			
	**F**	**df, ddf**	**p-value**	**R^2**	**F**	**df, ddf**	**p-value**	**R^2**	**F**	**df, ddf**	**p-value**	**R^2**
**Injury type**	7.2	3, 194	**< 0.001**	0.10	0.8	3, 55	0.520	0.04	0.0	4, 48	0.998	0.00
**Age**	11.8	1, 181	**< 0.001**	0.06	2.5	1, 60	0.120	0.04	0.2	1, 48	0.644	0.00
**Sex**	1.8	1, 157	0.177	0.01	0.0	1, 60	0.967	0.00	0.0	1, 45	0.953	0.00
**Fire severity**	0.5	4, 188	0.733	0.00	1.1	4,53	0.385	0.00	1.5	4, 43	0.215	0.04
**Rescue within fire zone**	0.0	1, 190	0.937	0.00	1.8	1, 56	0.181	0.03	2.2	1, 46	0.148	0.05
**Days since fire ignition when rescued**	1.7	1, 191	0.188	0.00	0.9	1, 56	0.342	0.00	1.7	1, 46	0.200	0.01

Table S9. Triage outcome for wildlife rescued with burns injuries via Kangaroo Island Wildlife Park Hospital

Species group	Euthanase	Hospitalised	Release	Not recorded	Total
Kangaroo	4	1			5
Koala	11	170	27	3	211
Possum	20				20
Wallaby	11				11
**Total**	**46**	**171**	**27**	**3**	**247**

Figure S1. Map of the eastern coast of NSW, Australia, depicting the location of bushfire-related marsupial reports in 2019/20 in relation to the fire extent.

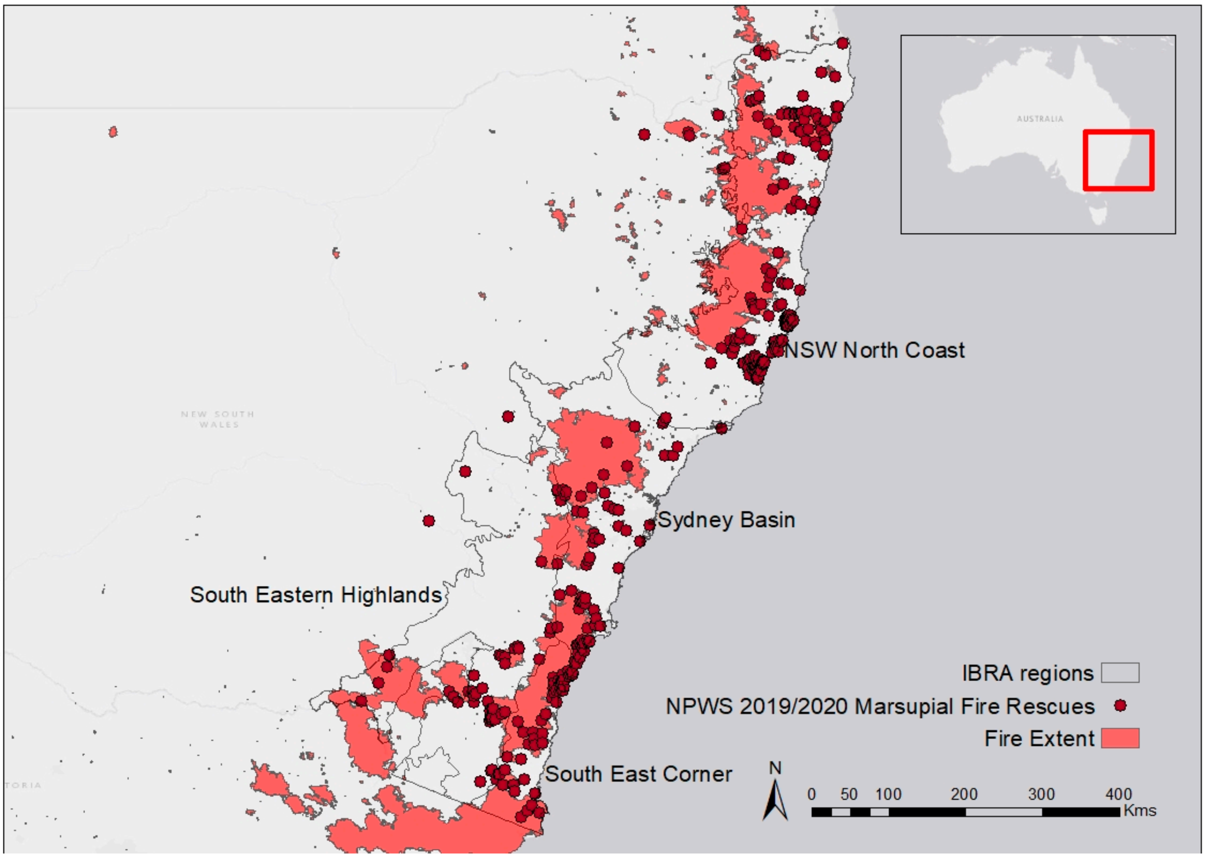

Supplement: Supplementary file 1 [file animals-14-01019-s001.zip › animals-2875817-supplementary.pdf]
